# Robust Speed Tracking Control for Future Electric Vehicles under Network-Induced Delay and Road Slope Variation

**DOI:** 10.3390/s22051787

**Published:** 2022-02-24

**Authors:** Jie Zhang, Qianrong Fan, Ming Wang, Bangji Zhang, Yuanchang Chen

**Affiliations:** 1 State Key Laboratory of Advanced Design and Manufacturing for Vehicle Body, College of Mechanical and Vehicle Engineering, Hunan University, Changsha 410082, China; jiezhang1906@hnu.edu.cn (J.Z.); qrfan@hnu.edu.cn (Q.F.); to_wm@hnu.edu.cn (M.W.); 2 Structural Dynamics and Acoustic Systems Laboratory, University of Massachusetts Lowell, One University Avenue, Lowell, MA 01854, USA; yuanchang_chen@student.uml.edu

**Keywords:** robust speed tracking control, integrated motor-transmission powertrain system, network-induced delay, parameter uncertainties, measurement noise, disturbance observer

## Abstract

Integrated motor-transmission (IMT) powertrain systems are widely used in future electric vehicles due to the advantages of their simple structure configuration and high controllability. In electric vehicles, precise speed tracking control is critical to ensure good gear shifting quality of an IMT powertrain system. However, the speed tracking control design becomes challenging due to the inevitable time delay of signal transmission introduced by the in-vehicle network and unknown road slope variation. Moreover, the system parameter uncertainties and signal measurement noise also increase the difficulty for the control algorithm. To address these issues, in this paper a robust speed tracking control strategy for electric vehicles with an IMT powertrain system is proposed. A disturbance observer and low-pass filter are developed to decrease the side effect from the unknown road slope variation and measurement noise and reduce the estimation error of the external load torque. Then, the network-induced delay speed tracking model is developed and is upgraded considering the damping coefficient uncertainties of the IMT powertrain system, which can be described through the norm-bounded uncertainty reduction method. To handle the network-induced delay and parameter uncertainties, a novel and less-conservative Lyapunov function is proposed to design the robust speed tracking controller by the linear matrix inequality (LMI) algorithm. Meanwhile, the estimation error and measurement noise are considered as the external disturbances in the controller design to promote robustness. Finally, the results demonstrate that the proposed controller has the advantages of strong robustness, excellent speed tracking performance, and ride comfort over the current existing controllers.

## 1. Introduction

Compared with traditional vehicles that use internal combustion engines as power sources, future electric vehicles (EVs) have higher economy, controllability, and intelligence [1,2]. As an advanced technology in the automotive industry, future EVs have attracted growing attention due to their significant advantages, which can be further developed as autonomous and intelligent vehicles. Since electric motors have the advantages of fast and accurate response to multiple working patterns, integrated motor-transmission powertrain system without a clutch has been widely installed in EVs [3]. This transmission configuration can provide high energy efficiency and smooth drivability. Due to the fast dynamic behavior and weak damping characteristics, the IMT powertrain system tends to have torsional oscillations [4,5]. It can deteriorate the vehicle maneuverability and ride comfort under complex disturbances, which commonly include road slope variation, signal transmission time delay in a vehicle control system, and measurement noise. Thus, speed tracking control has been a critical and challenging task for EVs.

Road condition variation and parameter uncertainties widely exist and increase the difficulty for the speed tracking control for EVs, bringing side effects to the vehicle system performance and stability [6,7]. To improve the whole system’s robustness, many studies have investigated reliable control methods against disturbance and uncertainties. For instance, Huang et al. [8] applied the differential-geometric approach and linear quadratic regulator (LQR) techniques to deal with multi-parametric uncertainties and to derive a nonlinear robust speed controller. Chu et al. [9] designed a hierarchical controller by using the LQR method to deal with an unmeasurable slope. Zhang et al. [10] roughly estimated the total vehicle mass and the road slope and then constructed a multiple layers estimator to further eliminate the estimation errors. Using a sliding-mode control (SMC) strategy based on fuzzy logic, Hu et al. [11] proposed a longitudinal controller for the autonomous ground vehicle with unknown nonlinearities and parametric uncertainties. Diba et al. [12] designed a robust proportional-integral (PI) torque controller and a proportional-integral-derivative (PID) speed controller by using the numerical optimization technique for the speed control of EV. Khooban et al. [13,14] used the nonlinear model predictive approach and the least-squares support vectors regression technique to deal with the nonlinearity in the EV system and designed corresponding speed controllers. Zhu et al. [15] presented a model predictive speed tracking control approach for autonomous ground vehicles, considering both model uncertainty and external disturbances. Vafamand et al. [16] developed a nonlinear model predictive controller (MPC) for the speed control of the constrained nonlinear electric vehicle, which was represented by linear parameter varying models with bias terms. Wang et al. [17] presented an output feedback robust controller-observer set using H∞ control theory to enhance the EV’s robust speed control performance. However, these published works ignore the interference from in-vehicle control systems, such as networked-induced delay (which may deteriorate the control quality).

To achieve acceptable speed tracking control performance and energy-saving demands, additional controllers and sensors are used in EVs, which increases the system time delay due to the limited in-vehicle network bandwidth [18]. The inevitable time delay can deteriorate the control performance or even lead to system instability [19,20,21]. Recently, many control approaches have been proposed to overcome the time delay problem. Considering the mixed H∞ and linear-quadratic regular performance, Liu et al. [22] designed a state-feedback controller for sample-data IMT powertrain systems that were connected via a controller area network (CAN). Luo et al. [23] dealt with the robust H∞ problem of a class of networked switched fuzzy systems with both network-induced delays and packet dropout using the multiple Lyapunov functions method. To reduce the conservatism of time-varying delay systems stability analysis, Lee et al. [24] suggested three novel Lyapunov functionals which were delay product type functions. Zeng et al. [25] proposed a generalized free-matrix-based inequality for the delay-dependent stability problem of a linear system with interval time-varying delays. The lower bound of the double functional integration was obtained more accurately and the positive definite condition was relaxed. However, the above studies are based on the assumption that the vehicle state information is accurately measured by in-vehicle sensors.

The vehicle speed tracking control greatly depends on the proper information of the real-time road condition and vehicle state in practical driving conditions. By using the vehicle sensor signals, the road condition and vehicle state information can be precisely estimated [26]. However, the measurement noise of the real sensors would reduce the accuracy of the vehicle state estimation. As described in [27,28,29], this can inevitably deteriorate the control of the dynamic performance of the whole vehicle system. To reduce the side effect of measurement noise, a low-pass filter is widely adopted in the design of the disturbance observer to achieve good observer performance [30,31,32]. Thus, a strong disturbance robustness observer is essential for the speed tracking control of EVs to obtain accurate information on the real-time road condition.

Granted, precise speed tracking control is an important technology to realize advanced assistant driving and automatic driving for future vehicles. Based on the above-mentioned descriptions, it is inevitably faced with these external disturbances, including network-induced delay, measurement noise and parameter uncertainties. However, most published works are focused mainly on dealing with one of these disturbances to achieve an acceptable control effect. To realize the precise speed control under complex working conditions, this paper simultaneously considers four typical disturbances to design a novel robust speed tracking control method for the IMT powertrain system is proposed in this work. The main contributions of this work are summarized below:

(1) Network-induced delay, parameter uncertainties, measurement noise and unknown road slope variation are simultaneously considered in the speed tracking controller design for the IMT powertrain system, which makes the controller more robust. There are a few works comprehensively addressing these control problems.
(2) A disturbance observer and low-pass filter are introduced to cope with the unknown road slope variation and measurement noise. Together, they can provide the accurate estimation value of external load torque for the controller.
(3) A norm-bounded uncertainty reduction method is employed to effectively describe the uncertainties of motor damping and driveshaft damping. Moreover, the time delay is explicitly handled by reconstructing a novel Lyapunov function. It can reduce the possible design conservativeness.
(4) Based on the constructed Lyapunov function, a novel robust speed tracking controller is proposed to achieve precise speed tracking performance and to reduce the oscillation of IMT powertrain system under these disturbances compared to the conventional controller.

The paper is organized as follows. In Section 2, the IMT powertrain model and vehicle speed tracking model are developed. The performance objectives are presented in Section 3. The robust vehicle speed tracking controller is designed in Section 4. Section 5 shows the comparative results under different disturbance conditions to verify the robustness and validity of the proposed controller. The paper is summarized in Section 6.

## 2. System Modeling

### 2.1. IMT Powertrain System Dynamics Model

**Figure 1:**
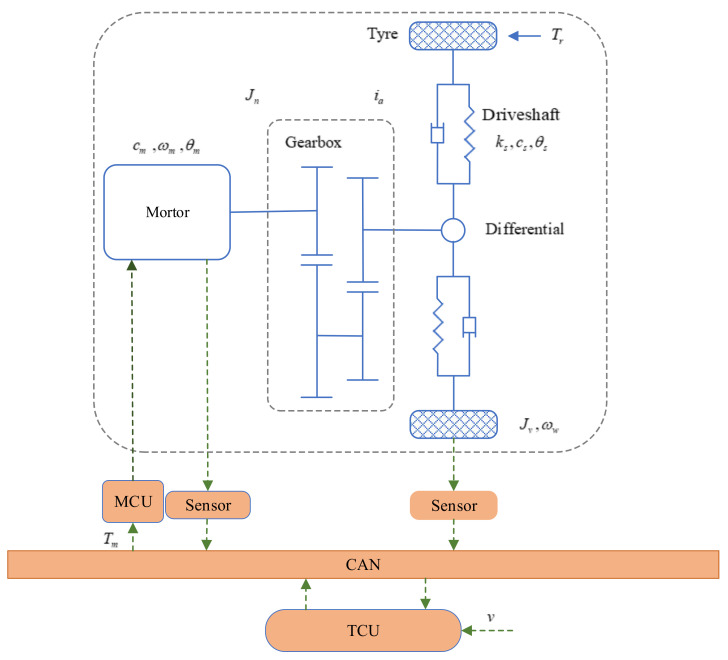
Schematic of IMT powertrain system.

The IMT powertrain system is schematically described in Figure 1. As shown in Figure 1, the electrified powertrain consists of a driving motor, a gearbox, a differential, and driveshafts. The signals among the sensors, torque control unit (TCU) and motor control unit (MCU) are transmitted through the CAN, which is usually used as the communication medium. The TCU can receive the signals of motor rotation speed, wheel rotation speed and the reference vehicle speed, and then calculate the motor torque, which will be sent to the MCU through CAN. The MCU drives the motor to provide the desired torque through the command from CAN [33].

The vehicle powertrain is simplified as lumped mass where the powertrain components are modeled as rigid bodies connected by the linear springs and linear dampers. By Newton’s second law, the equation of motion of the IMT powertrain system can be expressed by the following differential equations [33,34]:

(1) $$J_{n}{\dot{\omega }}_{m}=T_{m}-T_{s}/i_{a}-c_{m}\omega_{m}$$

(2) $$J_{v}\omega_{w}=T_{s}-T_{r}$$

(3) $$J_{v}=J_{w}+m_{v}r_{w}^{2}$$

(4) $$T_{s}=k_{s}\theta_{s}+c_{s}\left(\omega_{m}/i_{a}-\omega_{w}\right)$$

(5) $$\theta_{s}=\theta_{m}/i_{a}-\theta_{w}$$

(6) $$T_{r}=\mu m_{v}g\cos\left(\alpha \right)r_{w}+m_{v}g\sin\left(\alpha \right)r_{w}+0.5\rho_{air}A_{f}C_{d}\omega_{w}^{2}r_{w}^{3}$$

where $J_{n}$ denotes the composite inertia of the motor and gearbox, $\omega_{m}$ denotes the rotational speed of the motor, $T_{m}$ denotes the motor torque, $T_{s}$ denotes the driveshaft torque, $i_{a}$ denotes the total gear ratio of the powertrain, $c_{m}$ denotes the motor damping coefficient, $J_{v}$ denotes the equivalent inertia of the driveshaft, $J_{w}$ denotes the inertia of the wheel, $m_{v}$ denotes the vehicle mass, $r_{w}$ denotes the wheel radius, $\omega_{w}$ denotes the rotational speed of the wheel, $T_{r}$ denotes the external resistance load torque, $k_{s}$ denotes the stiffness coefficient of the driveshaft, $\theta_{s}$ denotes the torsional angle of the driveshaft, $\theta_{m}$ denotes the rotational displacement of the motor, $\theta_{w}$ denotes the rotational displacement of the wheel, $c_{s}$ denotes the damping coefficient of the driveshaft, μ denotes the rolling resistance coefficient, g denotes the gravitational acceleration, α denotes the road grade, $\rho_{air}$ denotes the air density, $A_{f}$ denotes the frontal area, and $C_{d}$ denotes the aerodynamic drag coefficient.

The state-space model of the IMT powertrain system can be written as:

(7) $$\dot{x}\left(t\right)=Ax\left(t\right)+Bu\left(t\right)+Dd\left(t\right)$$

In which $x={\left[\begin{array}{ccc} \omega_{m} & \omega_{w} & \theta_{s} \end{array}\right]}^{T}$ is the state variable, $u=T_{m}$ is the control input, $d={\left[\begin{array}{ccc} 0 & T_{r} & 0 \end{array}\right]}^{T}$ is the external disturbance. Based on Equation (1), the matrices A, B, and D are defined as:

(8) $$A=\left[\begin{array}{ccc} \frac{-\left(c_{s}/i_{a}^{2}+c_{m}\right)}{J_{n}} & \frac{c_{s}}{J_{n}\cdot i_{a}} & \frac{-k_{s}}{J_{n}\cdot i_{a}}\\ \frac{c_{s}}{J_{v}\cdot i_{a}} & \frac{-c_{s}}{J_{v}} & \frac{k_{s}}{J_{v}}\\ \frac{1}{i_{a}} & -1 & 0 \end{array}\right],\;B=\left[\begin{array}{c} \frac{1}{J_{n}}\\ 0\\ 0 \end{array}\right],\;D=\left[\begin{array}{ccc} 0 & 0 & 0\\ 0 & \frac{-1}{J_{v}} & 0\\ 0 & 0 & 0 \end{array}\right]$$

The motor rotational speed and wheel rotational speed can be measured by speed sensors with inevitable noise. Thus, the measured output of the IMT powertrain system can be described as:

(9) $$\;y\left(t\right)=Cx\left(t\right)+Cw\left(t\right)$$

where $y={\left[\omega_{m}\;\;\omega_{w}\;\;0\;\right]}^{T}$ is the measured output vector, $w={\left[\begin{array}{ccc} w_{1} & w_{2} & 0 \end{array}\right]}^{T}$ is the measurement noise, and $C=\left[\begin{array}{ccc} 1 & 0 & 0\\ 0 & 1 & 0\\ 0 & 0 & 0 \end{array}\right]$.

To calculate the matrix dimension in the controller design process, the variable $d\left(t\right)$ and $w\left(t\right)$, and the matrices C and D are zero-paddled to the appropriate dimensions.

**Remark** **1.**  *The external resistance load torque* $T_{r}$ *in the Equation (6) changes with rolling resistance coefficient* μ*, wheel rotational speed* $\omega_{w}$ *and road grade* α*, which is unavoidable in driving. Meanwhile, the complex nonlinear relationship between air drag torque and vehicle speed brings the troubles to the powertrain system modeling and controller design* [33]*. To estimate the actual resistance load torque, a disturbance estimator can be constructed as:*

(10) $${\hat{T}}_{r}=\left(T_{m}-c_{m}\omega_{m}-J_{n}{\dot{\omega }}_{m}\right)i_{a}-J_{v}{\dot{\omega }}_{w}$$

*where* ${\hat{T}}_{r}$ *is the estimated value of* $T_{r}$*, and* $T_{m}$ *can be collected from the MCU. The wheel and motor rotational speeds* $\omega_{w}$ *and* $\omega_{m}$ *can be measured from the sensors, and the corresponding rotational accelerations* ${\dot{\omega }}_{m}$ *and* ${\dot{\omega }}_{w}$ *can be obtained. Thus,* $T_{r}$ *can be estimated by Equation (10) without the time-varying parameter* α*. However, the measurement noises of* $\omega_{w}$ *and* $\omega_{m}$ *reduces the estimation accuracy of* $T_{r}$*. To solve this problem, a low-pass filter is adopted and the parameters of the filter will be discussed in Section 5.1.*

As the damping torque changes with the rotational speed and temperature, it is difficult to obtain a constant damping coefficient accurately. The norm-bounded uncertainty reduction method is introduced to address the uncertainties of the motor damping coefficient and driveshaft damping coefficient [19]. Thus, they can be expressed as

(11) $$c_{m}={\bar{c}}_{m}+\Delta c_{m}N_{1}$$

(12) $$c_{s}={\bar{c}}_{s}+\Delta c_{s}N_{2}$$

where $N_{i}(i=1,2)$ denote the time-varying parameters with satisfying $\left\|N_{i}\right\|\leq 1$, and ${\bar{c}}_{i}(i=m,s)$ denote the nominal values of $c_{i}$.

### 2.2. Vehicle Speed Tracking Model

The main objective of this work is to track the reference vehicle speed signal. Given a reference signal, the target motor rotational speed, wheel rotational speed, driveshaft torsional angle, and motor torque can be obtained as:

(13) $$\omega_{m}^{*}=i_{a}v^{*}/r_{w}$$

(14) $$\omega_{w}^{*}=v^{*}/r_{w}$$

(15) $$\theta_{s}^{*}=\left(J_{v}{\dot{\omega }}_{w}^{*}+T_{r}-{\bar{c}}_{s}\left(\omega_{m}^{*}/i_{a}-\omega_{w}^{*}\right)\right)/k_{s}$$

(16) $$T_{m}^{*}=T_{r}/i_{a}+{\bar{c}}_{m}i_{a}v^{*}/r_{w}+J_{v}{\dot{\omega }}_{w}^{*}/i_{a}+J_{n}{\dot{\omega }}_{m}^{*}$$

where $\omega_{m}^{*}$, $\omega_{w}^{*}$, $\theta_{s}^{*}$ and $T_{m}^{*}$ are the desired values of the corresponding variables, which are solved based on the above Equations (13)–(16) with any desired vehicle speed $v^{*}$. Defining the difference between the ideal value and the actual value of the motor torque, the speed tracking error model of the IMT powertrain system can be described as:

(17) $$\dot{\tilde{x}}\left(t\right)=\left(\bar{A}+\Delta A\right)\tilde{x}\left(t\right)+B\tilde{u}\left(t\right)+D\tilde{d}\left(t\right)$$

where $\tilde{x}={\left[{\tilde{x}}_{1}\;\;{\tilde{x}}_{2}\;\;{\tilde{x}}_{3}\right]}^{T}$ is the state variable of the speed tracking error model with ${\tilde{x}}_{1}\;=\omega_{m}-\omega_{m}^{*}$, ${\tilde{x}}_{2}\;=\omega_{w}-\omega_{w}^{*}$, and ${\tilde{x}}_{3}\;=\theta_{s}-\theta_{s}^{*}$. $\tilde{u}=T_{m}-T_{m}^{*}$ is the control input error. $\tilde{d}$ is the estimation error of $T_{r}$. The matrices $\bar{A}$ and ΔA are given as:

(18) $$\bar{A}=\left[\begin{array}{ccc} \frac{-\left({\bar{c}}_{s}/i_{a}^{2}+{\bar{c}}_{m}\right)}{J_{n}} & \frac{{\bar{c}}_{s}}{J_{n}\cdot i_{a}} & \frac{-k_{s}}{J_{n}\cdot i_{a}}\\ \frac{{\bar{c}}_{s}}{J_{v}\cdot i_{a}} & \frac{-{\bar{c}}_{s}}{J_{v}} & \frac{k_{s}}{J_{v}}\\ \frac{1}{i_{a}} & -1 & 0 \end{array}\right],\;\Delta A=EN\left(t\right)F$$

(19) $$E=\left[\begin{array}{ccc} \frac{-\left(\Delta c_{s}/i_{a}^{2}+\Delta c_{m}\right)}{J_{n}} & \frac{\Delta c_{s}}{J_{n}\cdot i_{a}} & 0\\ \frac{\Delta c_{s}}{J_{v}\cdot i_{a}} & \frac{-\Delta c_{s}}{J_{v}} & 0\\ 0 & 0 & 0 \end{array}\right],\;N\left(t\right)=\left[\begin{array}{ccc} N_{1}\left(t\right) & 0 & 0\\ 0 & N_{2}\left(t\right) & 0\\ 0 & 0 & N_{3}\left(t\right) \end{array}\right],\;F=I$$

Considering the time delay in the process of signal transmission, the output feedback controller is defined as follows:

(20) $$\tilde{y}\left(t\right)=C\tilde{x}\left(t-\tau_{sc}\right)+Cw\left(t\right)$$

(21) $$\tilde{u}\left(t\right)=K\tilde{y}\left(t\right)=KC\tilde{x}\left(t-\tau_{sc}-\tau_{ca}\right)+KCw\left(t\right)$$

where *K* is the control gain to be designed, $\tau_{sc}$ is the sensor-to-controller delay, and $\tau_{ac}$ is the controller-to-actuator delay.

The delay can be combined into a generalized form as:

(22) $$\tau \left(t\right)=\tau_{sc}+\tau_{ca}$$

Which then yields

(23) $$\dot{\tilde{x}}\left(t\right)=\left(\bar{A}+\Delta A\right)\tilde{x}\left(t\right)+BKC\tilde{x}\left(t-\tau \left(t\right)\right)+BKCw\left(t\right)+D\tilde{d}\left(t\right)$$

(24) $$0\leq \tau_{1}\leq \tau \left(t\right)\leq \tau_{2}$$

where, $\tau_{1}$ is the lower bound of the generalized delay and $\tau_{2}$ is the upper bound.

## 3. Performance Objective Formulation

To overcome the speed tracking issue, a robust controller is proposed to achieve precise tracking performance under different disturbances. The target vehicle speed is tracked by limiting the wheel speed tracking error. For this purpose, the wheel speed should be well controlled. Thus, the controlled output vector $z\left(t\right)$ can be defined as:

(25) $$z\left(t\right)=H\tilde{x}\left(t\right),\;H=[0\;\;1\;\;0]$$

The objective of the vehicle speed tracking control is to design a robust H∞ controller to ensure the asymptotical stability of the closed-loop system in Equation (23). The external resistance load torque estimation error $\tilde{d}\left(t\right)$ and measurement noise $w\left(t\right)$ can affect the speed tracking performance. Thus, a weighted robust H∞ performance is defined as follows:

(26) $$\int_{0}^{\infty }z^{T}\left(t\right)z\left(t\right)\leq \lambda^{2}\int_{0}^{\infty }w^{T}\left(t\right)w\left(t\right)+\kappa \lambda^{2}\int_{0}^{\infty }{\tilde{d}}^{T}\left(t\right)\tilde{d}\left(t\right)$$

where λ is the H∞ performance index and κ is the weighting factor.

## 4. Robust Vehicle Speed Tracking Controller Design

To have the required tracking performance of IMT powertrain systems, a robust controller is designed to handle the parameter uncertainties, measurement noise, load torque estimation error and network-induced delay. The development of the theorems of the controller is split into five lemmas as follows.

**Lemma** **1.** *For a positive definite matrix*R>0*and any continuously differentiable function *$x:\left[a,b\right]\to \mathbb{R}{\;}^{n}$*, the following equation holds* [35]*:*

(27) $$\int_{a}^{b}{\dot{x}}^{T}\left(s\right)R\dot{x}\left(s\right)ds\geq \frac{1}{h}\left[\Omega_{1}^{T}R\Omega_{1}+3\Omega_{2}^{T}R\Omega_{2}+5\Omega_{3}^{T}R\Omega_{3}\right]$$

*where*

(28) $$\Omega_{1}=x\left(b\right)-x\left(a\right),\;\Omega_{2}=x\left(b\right)+x\left(a\right)-\frac{2}{h}\int_{a}^{b}x\left(s\right)ds\;\mathrm{and}\;\Omega_{3}=x\left(b\right)-x\left(a\right)+\frac{6}{h}\int_{a}^{b}x\left(s\right)ds-\frac{12}{h^{2}}\int_{a}^{b}\int_{u}^{b}x\left(s\right)dsdu$$

**Lemma** **2.** *Let*$x:\left[a,b\right]\to \mathbb{R}{\;}^{n}$*be a continuously differentiable function. For a given symmetric positive definitive matrix *$R\in \mathbb{R}{\;}^{n\times n}$*and any matrices *$L_{1},\;L_{2}\in {\mathbb{R}}^{\;3n\times n}$*, the following equation holds* [36]*:*

(29) $$\int_{a}^{b}\int_{u}^{b}{\dot{x}}^{T}\left(s\right)R\dot{x}\left(s\right)dsdu\geq \pi^{T}\Omega \pi$$

*where*

(30) $$\begin{array}{l} \Omega =-\frac{1}{2}L_{1}R^{-1}L_{1}^{T}-\frac{1}{12}L_{2}R^{-1}L_{2}^{T}-sym\{L_{1}\left[\begin{array}{ccc} I & -I & 0 \end{array}\right]-L_{2}\left[\begin{array}{ccc} \frac{1}{2}I & I & -\frac{3}{2}I \end{array}\right]\},\\ \pi ={\left[x^{T}\left(b\right)\frac{1}{b-a}\int_{a}^{b}x^{T}\left(s\right)ds\frac{2}{{\left(b-a\right)}^{2}}\int_{a}^{b}\int_{u}^{b}x^{T}\left(s\right)dsdu\right]}^{T}. \end{array}$$

**Lemma** **3.** *For given positive integers* m *and* n *, a scalar* α *in the interval* (0, 1) *, a given matrix* R>0 *, and two matrices* $W_{1}$ *and* $W_{2}$ *in* $\mathbb{R}{\;}^{n\times m}$ *, the function* $\Xi \left(\alpha ,\mathbb{R}\right)$ *is defined for all vectors* ξ *in* $\mathbb{R}{\;}^{m}$ *by*

(31) $$\Xi \left(\alpha ,\mathbb{R}\right)=\frac{1}{\alpha }\xi^{T}W_{1}^{T}RW_{1}\xi +\frac{1}{1-\alpha }\xi^{T}W_{2}^{T}RW_{2}\xi$$

*Then, if there exists a matrix* X *in* $\mathbb{R}{\;}^{n\times n}$ *such that*

(32) $$\left[\begin{array}{cc} R & X\\ {}* & R \end{array}\right]\geq 0$$

*Then the following equation holds* [37]*:*

(33) $$\underset{\alpha \in \left(0,1\right)}{\min}\Xi \left(\alpha ,R\right)\geq {\left[\begin{array}{c} W_{1}\xi \\ W_{1}\xi \end{array}\right]}^{T}\left[\begin{array}{cc} R & X\\ {}* & R \end{array}\right]\left[\begin{array}{c} W_{1}\xi \\ W_{1}\xi \end{array}\right]$$

**Lemma** **4.** *If there exists a matrix*X*and positive definite matrix *W*, the following two conditions are equivalent* [38]*:*

(34) $$\left(W-X\right)W^{-1}\left(W-X\right)\geq 0$$

(35) $$-XW^{-1}X\leq W-2X$$

**Lemma** **5.** *Given matrices*E*, *$N\left(t\right)$*and *F*of appropriate dimensions, with *$N\left(t\right)$*satisfying *$N^{T}\left(t\right)N\left(t\right)\leq I$*, for any *μ>0*, the following equation holds* [39]*:*

(36) $$EN\left(t\right)F+{\left(EN\left(t\right)F\right)}^{T}\leq \mu^{-1}EE^{T}+\mu F^{T}F$$

The two following theorems are provided to guarantee that the system in Equation (23) is robustly asymptotically stable with a H∞ performance. Theorem 1 gives the controller stability condition with the given H∞ performance under time delay, measurement noise and load torque estimation error. Furthermore, Theorem 2 handles the problem of parameter uncertainties and converts the results into the LMIs form.

**Theorem** **1.** *For given scalars* $\tau_{1},\tau_{2},\kappa$ *, the system in Equation (23) is asymptotically stable and can satisfy the H∞ performance index (26), if there exist symmetric matrices* $0<P,Q_{i},R_{j}\in {\mathbb{R}}^{\;3\times 3}$ *,* i=1,2; j=1,2,3,4 *, scalar* λ>0 *and any matrices* $N_{k},M_{k},L_{k}\in {\mathbb{R}}^{\;9\times 3}$ *,* k=1,2 *,* $X\in \mathbb{R}{\;}^{9\times 9}$ *such that the following matrix inequalities hold:*

(37) $$\left[\begin{array}{ccc} \Phi_{1}+sym\{e_{1}^{T}P\Delta Ae_{1}\} & \bar{\Theta } & E_{A}^{T}P\\ {}* & \overset{⌢}{R} & 0\\ {}* & * & \left(\tau_{1}^{2}R_{1}+\tau_{12}^{2}R_{2}+\frac{\tau_{1}^{2}}{2}R_{3}+\frac{\tau_{12}^{2}}{2}R_{4}\right)-2P \end{array}\right]<0$$

(38) $$\left[\begin{array}{cc} \begin{array}{ccc} R_{2}+R_{4} & 0 & 0\\ {}* & 3\left(R_{2}+R_{4}\right) & 0\\ {}* & * & 5\left(R_{2}+R_{4}\right) \end{array} & X\\ {}* & \begin{array}{ccc} R_{2} & 0 & 0\\ {}* & 3R_{2} & 0\\ {}* & * & 5R_{2} \end{array} \end{array}\right]>0$$

*where*

(39) $$e_{s}=\left[\begin{array}{ccc} 0_{3\times \left(s-1\right)3} & I_{3\times 3} & 0_{3\times \left(12-s\right)3} \end{array}\right],\;s=1,2,\cdots ,12$$

(40) $$\begin{array}{l} \Phi_{1}=sym\{e_{1}^{T}\left[\begin{array}{cccc} P\bar{A} & PBKC & PBKC & PD \end{array}\right]{\bar{E}}_{1}\}+sym\{e_{1}^{T}P\Delta Ae_{1}\}+e_{1}^{T}H^{T}He_{1}-e_{11}^{T}\lambda^{2}Ie_{11}-e_{12}^{T}\kappa \lambda^{2}e_{12}+{\bar{E}}_{2}\bar{Q}{\bar{E}}_{2}^{T}\\ -{\left(e_{1}-e_{2}\right)}^{T}R_{1}\left(e_{1}-e_{2}\right)-3{\left(e_{1}+e_{2}-2e_{5}\right)}^{T}R_{1}\left(e_{1}+e_{2}-2e_{5}\right)-5{\left(e_{1}-e_{2}+6e_{5}-6e_{8}\right)}^{T}R_{1}\left(e_{1}-e_{2}+6e_{5}-6e_{8}\right)\\ +\Theta_{1}^{T}\left(N_{1}\Lambda_{1}-N_{2}\Lambda_{2}\right)\Theta_{1}+\Theta_{2}^{T}\left(M_{1}\Lambda_{3}-M_{2}\Lambda_{4}\right)\Theta_{2}+\Theta_{3}^{T}\left(L_{1}\Lambda_{5}-L_{2}\Lambda_{6}\right)\Theta_{3}-{\bar{\zeta }}_{1}^{T}{\bar{R}}_{1}{\bar{\zeta }}_{1}+{\bar{\zeta }}_{1}^{T}{\bar{R}}_{1}{\bar{\zeta }}_{1} \end{array}$$

(41) $$E_{A}=\left(\bar{A}+\Delta A\right)e_{1}+BKCe_{3}+BKCe_{11}+De_{12},\;\;{\bar{E}}_{1}={\left[\begin{array}{cccc} e_{1}^{T} & e_{3}^{T} & e_{11}^{T} & e_{12}^{T} \end{array}\right]}^{T},\;\;{\bar{E}}_{2}=\left[\begin{array}{ccc} e_{1}^{T} & e_{2}^{T} & e_{4}^{T} \end{array}\right]$$

(42) $$\bar{\Theta }=\left[\Theta_{1}^{T}N_{1},\Theta_{1}^{T}N_{2},\Theta_{2}^{T}M_{1},\Theta_{2}^{T}M_{2},\Theta_{3}^{T}L_{1},\Theta_{3}^{T}L_{2}\right],\;\;\bar{Q}==diag\left\{Q_{1},\;Q_{2}-Q_{1},\;Q_{2}\right\}$$

(43) $$\overset{⌢}{R}=diag\{-2R_{3},-12R_{3},-2R_{4},-12R_{4},-2R_{4},-12R_{4}\},\;\tau_{12}=\tau_{2}-\tau_{1}$$

(44) $$\Theta_{1}={\left[\begin{array}{ccc} e_{1}^{T} & e_{5}^{T} & e_{8}^{T} \end{array}\right]}^{T},\;\;\Theta_{2}={\left[\begin{array}{ccc} e_{2}^{T} & e_{6}^{T} & e_{9}^{T} \end{array}\right]}^{T},\;\;\Theta_{3}={\left[\begin{array}{ccc} e_{3}^{T} & e_{7}^{T} & e_{10}^{T} \end{array}\right]}^{T}$$

(45) $$\Lambda_{1}=\left(e_{1}-e_{5}\right),\;\;\Lambda_{2}=\left(\frac{1}{2}e_{1}+e_{5}-\frac{3}{2}e_{8}\right),\;\;\Lambda_{3}=\left(e_{2}-e_{6}\right),\;\;\Lambda_{4}=\left(\frac{1}{2}e_{2}+e_{6}-\frac{3}{2}e_{9}\right)$$

(46) $$\Lambda_{5}=\left(e_{3}-e_{7}\right),\;\;\Lambda_{6}=\left(\frac{1}{2}e_{3}+e_{7}-\frac{3}{2}e_{10}\right),\;\;\zeta_{1}=\left[e_{2}-e_{3}\right]$$

**Proof.**  Define a new Lyapunov function for the system in Equation (23) as

(47) $$V\left(t\right)=V_{1}\left(t\right)+V_{2}\left(t\right)+V_{3}\left(t\right)+V_{4}\left(t\right)+V_{5}\left(t\right)+V_{6}\left(t\right)$$

where

(48) $$\begin{array}{l} V_{1}\left(t\right)={\tilde{x}}^{T}\left(t\right)P\tilde{x}\left(t\right),\;V_{2}\left(t\right)=\int_{t-\tau_{1}}^{t}{\tilde{x}}^{T}\left(s\right)Q_{1}\tilde{x}\left(s\right)ds+\int_{t-\tau_{2}}^{t-\tau_{1}}{\tilde{x}}^{T}\left(s\right)Q_{2}\tilde{x}\left(s\right)ds,\\ V_{3}\left(x_{t}\right)=\tau_{1}\int_{-\tau_{1}}^{0}\int_{t+\lambda }^{t}{\dot{\tilde{x}}}^{T}\left(s\right)R_{1}\dot{\tilde{x}}\left(s\right)dsd\lambda ,\;V_{4}\left(x_{t}\right)=\tau_{12}\int_{-\tau_{2}}^{-\tau_{1}}\int_{t+\lambda }^{t}{\dot{\tilde{x}}}^{T}\left(s\right)R_{2}\dot{\tilde{x}}\left(s\right)dsd\lambda ,\\ V_{5}\left(x_{t}\right)=\int_{-\tau_{1}}^{0}\int_{\alpha }^{0}\int_{t+\lambda }^{t}{\dot{\tilde{x}}}^{T}\left(s\right)R_{3}\dot{\tilde{x}}\left(s\right)dsd\lambda d\alpha ,\;V_{6}\left(x_{t}\right)=\int_{-\tau_{2}}^{-\tau_{1}}\int_{\alpha }^{-\tau_{1}}\int_{t+\lambda }^{t}{\dot{\tilde{x}}}^{T}\left(s\right)R_{4}\dot{\tilde{x}}\left(s\right)dsd\lambda d\alpha , \end{array}$$

in which $P,{\;Q}_{1}{,\;Q}_{2},{\;R}_{1},{\;R}_{2},{\;R}_{3}{\;\mathrm{and}\;R}_{4}$ are the symmetric positive definite matrices.

An augmented vector $\xi \left(t\right)$ is defined as:

(49) $$\xi \left(t\right)={\left[\eta_{1}^{T}\left(t\right)\;\eta_{2}^{T}\left(t\right)\;\eta_{3}^{T}\left(t\right)\;\eta_{4}^{T}\left(t\right)\;\eta_{5}^{T}\left(t\right)\;\eta_{6}^{T}\left(t\right)\;\eta_{7}^{T}\left(t\right)\;\eta_{8}^{T}\left(t\right)\;\eta_{9}^{T}\left(t\right)\;\eta_{10}^{T}\left(t\right)\;\eta_{11}^{T}\left(t\right)\;\eta_{12}^{T}\left(t\right)\right]}^{T}$$

where

(50) $$\eta_{1}^{T}\left(t\right)={\tilde{x}}^{T}\left(t\right),\;\eta_{2}^{T}\left(t\right)={\tilde{x}}^{T}\left(t-\tau_{1}\right),\;\eta_{3}^{T}\left(t\right)={\tilde{x}}^{T}\left(t-\tau \left(t\right)\right),\;\eta_{4}^{T}\left(t\right)={\tilde{x}}^{T}\left(t-\tau_{2}\right)$$

(51) $$\eta_{5}^{T}\left(t\right)=\frac{1}{\tau_{1}}\int_{t-\tau_{1}}^{t}{\tilde{x}}^{T}\left(s\right)ds,\;\eta_{6}^{T}\left(t\right)=\frac{1}{\tau \left(t\right)-\tau_{1}}\int_{t-\tau \left(t\right)}^{t-\tau_{1}}{\tilde{x}}^{T}\left(s\right)ds,\;\eta_{7}^{T}\left(t\right)=\frac{1}{\tau_{2}-\tau \left(t\right)}\int_{t-\tau_{2}}^{t-\tau \left(t\right)}{\tilde{x}}^{T}\left(s\right)ds$$

(52) $$\eta_{8}^{T}\left(t\right)=\frac{2}{\tau_{1}^{2}}\int_{-\tau_{1}}^{0}\int_{t+\lambda }^{t}{\tilde{x}}^{T}\left(s\right)dsd\lambda ,\;\eta_{9}^{T}\left(t\right)=\frac{2}{{\left(\tau \left(t\right)-\tau_{1}\right)}^{2}}\int_{-\tau \left(t\right)}^{-\tau_{1}}\int_{t+\lambda }^{t-\tau_{1}}{\tilde{x}}^{T}\left(s\right)dsd\lambda$$

(53) $$\eta_{10}^{T}\left(t\right)=\frac{2}{{\left(\tau_{2}-\tau \left(t\right)\right)}^{2}}\int_{-\tau_{2}}^{-\tau \left(t\right)}\int_{t+\lambda }^{t-\tau \left(t\right)}{\tilde{x}}^{T}\left(s\right)dsd\lambda ,\;\eta_{11}^{T}\left(t\right)=w^{T}\left(t\right),\;\eta_{12}^{T}\left(t\right)={\tilde{d}}^{T}\left(t\right)$$

Taking the time derivative of $V_{i}\left(t\right),\;i=1,\;2,\cdot \cdot \cdot ,\;6$ along the trajectories of the system (23), one can obtain:

(54) $$\dot{V}\left(t\right)={\dot{V}}_{1}\left(t\right)+{\dot{V}}_{2}\left(t\right)+{\dot{V}}_{3}\left(t\right)+{\dot{V}}_{4}\left(t\right)+{\dot{V}}_{5}\left(t\right)+{\dot{V}}_{6}\left(t\right)$$

(55) $${\dot{V}}_{1}\left(t\right)=2{\tilde{x}}^{T}\left(t\right)P\dot{\tilde{x}}\left(t\right)=sym\left\{\eta_{1}^{T}P\left(\left(\bar{A}+\Delta A\right)\eta_{1}+BKC\eta_{3}+BKC\eta_{11}+D\eta_{12}\right)\right\}$$

(56) $${\dot{V}}_{2}\left(t\right)=\eta_{1}^{T}Q_{1}\eta_{1}+\eta_{2}^{T}\left(Q_{2}-Q_{1}\right)\eta_{2}-\eta_{4}^{T}Q_{2}\eta_{4}$$

(57) $${\dot{V}}_{3}\left(t\right)=\tau_{1}^{2}{\dot{\tilde{x}}}^{T}\left(t\right)R_{1}\dot{\tilde{x}}\left(t\right)-\tau_{1}\int_{t-\tau_{1}}^{t}{\dot{\tilde{x}}}^{T}\left(s\right)R_{1}\dot{\tilde{x}}\left(s\right)ds$$

(58) $${\dot{V}}_{4}\left(t\right)=\tau_{12}^{2}{\dot{\tilde{x}}}^{T}\left(t\right)R_{2}\dot{\tilde{x}}\left(t\right)-\tau_{12}\int_{t-\tau_{2}}^{t-\tau_{1}}{\dot{\tilde{x}}}^{T}\left(s\right)R_{2}\dot{\tilde{x}}\left(s\right)ds$$

(59) $${\dot{V}}_{5}\left(t\right)=\frac{\tau_{1}^{2}}{2}{\dot{\tilde{x}}}^{T}\left(t\right)R_{3}\dot{\tilde{x}}\left(t\right)-\int_{-\tau_{1}}^{0}\int_{t+\lambda }^{t}{\dot{\tilde{x}}}^{T}\left(s\right)R_{3}\dot{\tilde{x}}\left(s\right)dsd\lambda$$

(60) $${\dot{V}}_{6}\left(t\right)=\frac{\tau_{12}^{2}}{2}{\dot{\tilde{x}}}^{T}\left(t\right)R_{4}\dot{\tilde{x}}\left(t\right)-\int_{-\tau_{2}}^{-\tau_{1}}\int_{t+\lambda }^{t-\tau_{1}}{\dot{\tilde{x}}}^{T}\left(s\right)R_{4}\dot{\tilde{x}}\left(s\right)dsd\lambda$$

For the integral terms on the Equation (57), one has the following condition by using Lemma 1

(61) $$\begin{array}{ll} -\tau_{1}\int_{t-\tau_{1}}^{t}{\dot{\tilde{x}}}^{T}\left(s\right)R_{1}\dot{\tilde{x}}\left(s\right)ds & \leq -\xi^{T}\left(t\right)\{{\left(e_{1}-e_{2}\right)}^{T}R_{1}\left(e_{1}-e_{2}\right)+3{\left(e_{1}+e_{2}-2e_{5}\right)}^{T}R_{1}\left(e_{1}+e_{2}-2e_{5}\right)\\ & +5{\left(e_{1}-e_{2}+6e_{5}-6e_{8}\right)}^{T}R_{1}\left(e_{1}-e_{2}+6e_{5}-6e_{8}\right)\}\xi \left(t\right). \end{array}$$

According to Lemma 2, for the double integral terms on the Equation (59), one has:

(62) $$\begin{array}{ll} -\int_{-\tau_{1}}^{0}\int_{t+\lambda }^{t}{\dot{\tilde{x}}}^{T}\left(s\right)R_{3}\dot{\tilde{x}}\left(s\right)dsd\lambda & \leq \xi^{T}\left(t\right)\Theta_{1}^{T}\left(\frac{1}{2}N_{1}R_{3}^{-1}N_{1}^{T}+\frac{1}{12}N_{2}R_{3}^{-1}N_{2}^{T}\right)\Theta_{1}\xi \left(t\right)\\ & +\xi^{T}\left(t\right)\Theta_{1}^{T}\left(N_{1}\Lambda_{1}-N_{2}\Lambda_{2}\right)\Theta_{1}\xi \left(t\right). \end{array}$$

To dispose of the integral limit in the integral terms of (58) and (60), the integral interval is split as the following forms, respectively

(63) $$-\tau_{12}\int_{t-\tau_{2}}^{t-\tau_{1}}{\dot{\tilde{x}}}^{T}\left(s\right)R_{2}\dot{\tilde{x}}\left(s\right)ds=-\tau_{12}\int_{t-\tau \left(t\right)}^{t-\tau_{1}}{\dot{\tilde{x}}}^{T}\left(s\right)R_{2}\dot{\tilde{x}}\left(s\right)ds-\tau_{12}\int_{t-\tau_{2}}^{t-\tau \left(t\right)}{\dot{\tilde{x}}}^{T}\left(s\right)R_{2}\dot{\tilde{x}}\left(s\right)ds$$

(64) $$\begin{array}{c} -\int_{-\tau_{2}}^{-\tau_{1}}\int_{t+\lambda }^{t-\tau_{1}}{\dot{\tilde{x}}}^{T}\left(s\right)R_{4}\dot{\tilde{x}}\left(s\right)dsd\lambda =-\int_{-\tau \left(t\right)}^{-\tau_{1}}\int_{t+\lambda }^{t-\tau_{1}}{\dot{\tilde{x}}}^{T}\left(s\right)R_{4}\dot{\tilde{x}}\left(s\right)dsd\lambda -\int_{-\tau_{2}}^{-\tau \left(t\right)}\int_{t+\lambda }^{t-\tau \left(t\right)}{\dot{\tilde{x}}}^{T}\left(s\right)R_{4}\dot{\tilde{x}}\left(s\right)dsd\lambda \\ -\left(\tau_{2}-\tau \left(t\right)\right)\int_{t-\tau \left(t\right)}^{t-\tau_{1}}{\dot{\tilde{x}}}^{T}\left(s\right)R_{4}\dot{\tilde{x}}\left(s\right)dsd\lambda . \end{array}$$

The single integral terms in the Equations (63) and (64) can be formulated as:

(65) $$\begin{array}{cc} \; & -\tau_{12}\int_{t-\tau \left(t\right)}^{t-\tau_{1}}{\dot{\tilde{x}}}^{T}\left(s\right)R_{2}\dot{\tilde{x}}\left(s\right)ds-\tau_{12}\int_{t-\tau_{2}}^{t-\tau \left(t\right)}{\dot{\tilde{x}}}^{T}\left(s\right)R_{2}\dot{\tilde{x}}\left(s\right)ds-\left(\tau_{2}-\tau \left(t\right)\right)\int_{t-\tau \left(t\right)}^{t-\tau_{1}}{\dot{\tilde{x}}}^{T}\left(s\right)R_{4}\dot{\tilde{x}}\left(s\right)ds\\ \; & =\frac{\tau_{12}}{\tau \left(t\right)-\tau_{1}}\left(-\left(\tau \left(t\right)-\tau_{1}\right)\int_{t-\tau \left(t\right)}^{t-\tau_{1}}{\dot{\tilde{x}}}^{T}\left(s\right)R_{2}\dot{\tilde{x}}\left(s\right)ds\right)+\frac{\tau_{12}}{\tau_{2}-\tau \left(t\right)}\left(-\left(\tau_{2}-\tau \left(t\right)\right)\int_{t-\tau_{2}}^{t-\tau \left(t\right)}{\dot{\tilde{x}}}^{T}\left(s\right)R_{2}\dot{\tilde{x}}\left(s\right)ds\right)\\ \; & +\frac{\tau_{2}-\tau \left(t\right)}{\tau \left(t\right)-\tau_{1}}\left(-\left(\tau \left(t\right)-\tau_{1}\right)\int_{t-\tau \left(t\right)}^{t-\tau_{1}}{\dot{\tilde{x}}}^{T}\left(s\right)R_{4}\dot{\tilde{x}}\left(s\right)ds\right)\\ \; & =\frac{1}{\alpha }\left(-\left(\tau \left(t\right)-\tau_{1}\right)\int_{t-\tau \left(t\right)}^{t-\tau_{1}}{\dot{\tilde{x}}}^{T}\left(s\right)R_{2}\dot{\tilde{x}}\left(s\right)ds\right)+\frac{1}{1-\alpha }\left(-\left(\tau_{2}-\tau \left(t\right)\right)\int_{t-\tau_{2}}^{t-\tau \left(t\right)}{\dot{\tilde{x}}}^{T}\left(s\right)R_{2}\dot{\tilde{x}}\left(s\right)ds\right)\\ \; & +\left(\frac{1}{\alpha }-1\right)\left(-\left(\tau \left(t\right)-\tau_{1}\right)\int_{t-\tau \left(t\right)}^{t-\tau_{1}}{\dot{\tilde{x}}}^{T}\left(s\right)R_{4}\dot{\tilde{x}}\left(s\right)ds\right), \end{array}$$

where α is the positive scalar. Based on Lemma 1, one has:

(66) $$\begin{array}{cc} \; & -\tau_{12}\int_{t-\tau \left(t\right)}^{t-\tau_{1}}{\dot{\tilde{x}}}^{T}\left(s\right)R_{2}\dot{\tilde{x}}\left(s\right)ds-\tau_{12}\int_{t-\tau_{2}}^{t-\tau \left(t\right)}{\dot{\tilde{x}}}^{T}\left(s\right)R_{2}\dot{\tilde{x}}\left(s\right)ds-\left(\tau_{2}-\tau \left(t\right)\right)\int_{t-\tau \left(t\right)}^{t-\tau_{1}}{\dot{\tilde{x}}}^{T}\left(s\right)R_{4}\dot{\tilde{x}}\left(s\right)ds\\ \; & \leq -\frac{1}{\alpha }\xi^{T}\left(t\right){\left[\begin{array}{c} \zeta_{1}\\ \zeta_{2}\\ \zeta_{3} \end{array}\right]}^{T}\left[\begin{array}{ccc} R_{2} & 0 & 0\\ {}* & 3R_{2} & 0\\ {}* & * & 5R_{2} \end{array}\right]\left[\begin{array}{c} \zeta_{1}\\ \zeta_{2}\\ \zeta_{3} \end{array}\right]\xi \left(t\right)-\frac{1}{1-\alpha }\xi^{T}\left(t\right){\left[\begin{array}{c} \zeta_{4}\\ \zeta_{5}\\ \zeta_{6} \end{array}\right]}^{T}\left[\begin{array}{ccc} R_{2} & 0 & 0\\ {}* & 3R_{2} & 0\\ {}* & * & 5R_{2} \end{array}\right]\left[\begin{array}{c} \zeta_{4}\\ \zeta_{5}\\ \zeta_{6} \end{array}\right]\xi \left(t\right)\\ \; & -\left(\frac{1}{\alpha }-1\right)\xi^{T}\left(t\right){\left[\begin{array}{c} \zeta_{1}\\ \zeta_{2}\\ \zeta_{3} \end{array}\right]}^{T}\left[\begin{array}{ccc} R_{4} & 0 & 0\\ {}* & 3R_{4} & 0\\ {}* & * & 5R_{4} \end{array}\right]\left[\begin{array}{c} \zeta_{1}\\ \zeta_{2}\\ \zeta_{3} \end{array}\right]\xi \left(t\right)\\ \; & \leq -\frac{1}{\alpha }\xi^{T}\left(t\right){\left[\begin{array}{c} \zeta_{1}\\ \zeta_{2}\\ \zeta_{3} \end{array}\right]}^{T}\left[\begin{array}{ccc} R_{2}+R_{4} & 0 & 0\\ {}* & 3\left(R_{2}+R_{4}\right) & 0\\ {}* & * & 5\left(R_{2}+R_{4}\right) \end{array}\right]\left[\begin{array}{c} \zeta_{1}\\ \zeta_{2}\\ \zeta_{3} \end{array}\right]\xi \left(t\right)\\ \; & -\frac{1}{1-\alpha }\xi^{T}\left(t\right){\left[\begin{array}{c} \zeta_{4}\\ \zeta_{5}\\ \zeta_{6} \end{array}\right]}^{T}\left[\begin{array}{ccc} R_{2} & 0 & 0\\ {}* & 3R_{2} & 0\\ {}* & * & 5R_{2} \end{array}\right]\left[\begin{array}{c} \zeta_{4}\\ \zeta_{5}\\ \zeta_{6} \end{array}\right]\xi \left(t\right)+\xi^{T}\left(t\right){\left[\begin{array}{c} \zeta_{1}\\ \zeta_{2}\\ \zeta_{3} \end{array}\right]}^{T}\left[\begin{array}{ccc} R_{4} & 0 & 0\\ {}* & 3R_{4} & 0\\ {}* & * & 5R_{4} \end{array}\right]\left[\begin{array}{c} \zeta_{1}\\ \zeta_{2}\\ \zeta_{3} \end{array}\right]\xi \left(t\right), \end{array}$$

with

$$\zeta_{1}=\left[e_{2}-e_{3}\right],\zeta_{2}=\left[e_{2}+e_{3}-2e_{6}\right],\zeta_{3}=\left[e_{2}-e_{3}+6e_{6}-6e_{9}\right],\zeta_{4}=\left[e_{3}-e_{4}\right],\zeta_{5}=\left[e_{3}+e_{4}-2e_{7}\right],\zeta_{6}=\left[e_{3}-e_{4}+6e_{7}-6e_{10}\right].$$

Using Lemma 3 in Equation (66), one has:

(67) $$\begin{array}{l} -\tau_{12}\int_{t-\tau \left(t\right)}^{t-\tau_{1}}{\dot{\tilde{x}}}^{T}\left(s\right)R_{2}\dot{\tilde{x}}\left(s\right)ds-\tau_{12}\int_{t-\tau_{2}}^{t-\tau \left(t\right)}{\dot{\tilde{x}}}^{T}\left(s\right)R_{2}\dot{\tilde{x}}\left(s\right)ds-\left(\tau_{2}-\tau \left(t\right)\right)\int_{t-\tau \left(t\right)}^{t-\tau_{1}}{\dot{\tilde{x}}}^{T}\left(s\right)R_{4}\dot{\tilde{x}}\left(s\right)ds\\ \leq -\xi^{T}\left(t\right){\bar{\zeta }}_{1}^{T}{\bar{R}}_{1}{\bar{\zeta }}_{1}\xi \left(t\right)+\xi^{T}\left(t\right){\bar{\zeta }}_{2}^{T}{\bar{R}}_{2}{\bar{\zeta }}_{2}\xi \left(t\right), \end{array}$$

in which

(68) $$\begin{array}{l} {\bar{\zeta }}_{1}={\left[\begin{array}{cccccc} \zeta_{1}^{T} & \zeta_{2}^{T} & \zeta_{3}^{T} & \zeta_{4}^{T} & \zeta_{5}^{T} & \zeta_{6}^{T} \end{array}\right]}^{T},{\bar{\zeta }}_{2}={\left[\begin{array}{ccc} \zeta_{1}^{T} & \zeta_{2}^{T} & \zeta_{3}^{T} \end{array}\right]}^{T},\\ {\bar{R}}_{1}=\left[\begin{array}{cc} \begin{array}{ccc} R_{2}+R_{4} & 0 & 0\\ {}* & 3\left(R_{2}+R_{4}\right) & 0\\ {}* & * & 5\left(R_{2}+R_{4}\right) \end{array} & X\\ {}* & \begin{array}{ccc} R_{2} & 0 & 0\\ {}* & 3R_{2} & 0\\ {}* & * & 5R_{2} \end{array} \end{array}\right]>0,{\bar{R}}_{2}=\left[\begin{array}{ccc} R_{4} & 0 & 0\\ {}* & 3R_{4} & 0\\ {}* & * & 5R_{4} \end{array}\right]. \end{array}$$

Thus, the condition (38) can be obtained.

For the double integral terms on Equation (64), by using Lemma 2, one has:

(69) $$\begin{array}{ll} -\int_{-\tau \left(t\right)}^{-\tau_{1}}\int_{t+\lambda }^{t-\tau_{1}}{\dot{\tilde{x}}}^{T}\left(s\right)R_{4}\dot{\tilde{x}}\left(s\right)dsd\lambda & \leq \xi^{T}\left(t\right)\Theta_{2}^{T}\left(\frac{1}{2}M_{1}R_{4}^{-1}M_{1}^{T}+\frac{1}{12}M_{2}R_{4}^{-1}M_{2}^{T}\right)\Theta_{2}\xi \left(t\right)\\ & +\xi^{T}\left(t\right)\Theta_{2}^{T}\left(M_{1}\Lambda_{3}-M_{2}\Lambda_{4}\right)\Theta_{2}\xi \left(t\right),\\ -\int_{-\tau_{2}}^{-\tau \left(t\right)}\int_{t+\lambda }^{t-\tau \left(t\right)}{\dot{\tilde{x}}}^{T}\left(s\right)R_{4}\dot{\tilde{x}}\left(s\right)dsd\lambda & \leq \xi^{T}\left(t\right)\Theta_{3}^{T}\left(\frac{1}{2}L_{1}R_{4}^{-1}L_{1}^{T}+\frac{1}{12}L_{2}R_{4}^{-1}L_{2}^{T}\right)\Theta_{3}\xi \left(t\right)\\ & +\xi^{T}\left(t\right)\Theta_{3}^{T}\left(L_{1}\Lambda_{5}-L_{2}\Lambda_{6}\right)\Theta_{3}\xi \left(t\right). \end{array}$$

By adding $z{\left(t\right)}^{T}z-\lambda^{2}w^{T}\left(t\right)w\left(t\right)-\kappa \lambda^{2}{\tilde{d}}^{T}\left(t\right)\tilde{d}\left(t\right)$ with λ>0, κ>0 to both sides of the Equation (54), and combing (61)–(69) together, we can obtain:

(70) $$\begin{array}{cc} \; & J=\dot{V}\left(t\right)+z{\left(t\right)}^{T}z-\lambda^{2}w^{T}\left(t\right)w\left(t\right)-\kappa \lambda^{2}{\tilde{d}}^{T}\left(t\right)\tilde{d}\left(t\right)\\ \; & \leq \xi^{T}\left(t\right)\;\;\;\{\;\;\;sym\;\;\;\left\{\;\;\;e_{1}^{T}\left[\begin{array}{cccc} P\bar{A} & PBKC & PBKC & PD \end{array}\right]{\bar{E}}_{1}+e_{1}^{T}P\Delta Ae_{1}\;\;\;\right\}\;\;\;+e_{1}^{T}H^{T}He_{1}-e_{11}^{T}\lambda^{2}Ie_{11}-e_{12}^{T}\kappa \lambda^{2}e_{12}\\ \; & +{\bar{E}}_{2}\bar{Q}{\bar{E}}_{2}^{T}-{\left(e_{1}-e_{2}\right)}^{T}R_{1}\left(e_{1}-e_{2}\right)-3{\left(e_{1}+e_{2}-2e_{5}\right)}^{T}R_{1}\left(e_{1}+e_{2}-2e_{5}\right)\\ \; & -5{\left(e_{1}-e_{2}+6e_{5}-6e_{8}\right)}^{T}R_{1}\left(e_{1}-e_{2}+6e_{5}-6e_{8}\right)+\Theta_{1}^{T}\left(\frac{1}{2}N_{1}R_{3}^{-1}N_{1}^{T}+\frac{1}{12}N_{2}R_{3}^{-1}N_{2}^{T}\right)\Theta_{1}\\ \; & +\Theta_{1}^{T}\left(N_{1}\Lambda_{1}-N_{2}\Lambda_{2}\right)\Theta_{1}+\Theta_{2}^{T}\left(\frac{1}{2}M_{1}R_{4}^{-1}M_{1}^{T}+\frac{1}{12}M_{2}R_{4}^{-1}M_{2}^{T}\right)\Theta_{2}+\Theta_{2}^{T}\left(M_{1}\Lambda_{3}-M_{2}\Lambda_{4}\right)\Theta_{2}\\ \; & +\Theta_{3}^{T}\left(\frac{1}{2}L_{1}R_{4}^{-1}L_{1}^{T}+\frac{1}{12}L_{2}R_{4}^{-1}L_{2}^{T}\right)\Theta_{3}+\Theta_{3}^{T}\left(L_{1}\Lambda_{5}-L_{2}\Lambda_{6}\right)\Theta_{3}-{\bar{\zeta }}_{1}^{T}{\bar{R}}_{1}{\bar{\zeta }}_{1}+{\bar{\zeta }}_{1}^{T}{\bar{R}}_{1}{\bar{\zeta }}_{1}\\ \; & +E_{A}^{T}\left(\tau_{1}^{2}R_{1}+\tau_{12}^{2}R_{2}+\frac{\tau_{1}^{2}}{2}R_{3}+\frac{\tau_{12}^{2}}{2}R_{4}\right)E_{A}\;\;\;\}\;\;\;\xi \left(t\right). \end{array}$$

Then, the Equation (70) can be rewritten as:

(71) $$J\left(t\right)\leq \xi^{T}\left(t\right)\{\Phi_{1}+\Phi_{2}\}\xi \left(t\right)$$

where,

(72) $$\begin{array}{l} \Phi_{2}=\Theta_{1}^{T}\left(\frac{1}{2}N_{1}R_{3}^{-1}N_{1}^{T}+\frac{1}{12}N_{2}R_{3}^{-1}N_{2}^{T}\right)\Theta_{1}+\Theta_{2}^{T}\left(\frac{1}{2}M_{1}R_{4}^{-1}M_{1}^{T}+\frac{1}{12}M_{2}R_{4}^{-1}M_{2}^{T}\right)\Theta_{2}+\Theta_{3}^{T}\left(\frac{1}{2}L_{1}R_{4}^{-1}L_{1}^{T}+\frac{1}{12}L_{2}R_{4}^{-1}L_{2}^{T}\right)\Theta_{3}\\ +E_{A}^{T}\left(\tau_{1}^{2}R_{1}+\tau_{12}^{2}R_{2}+\frac{\tau_{1}^{2}}{2}R_{3}+\frac{\tau_{12}^{2}}{2}R_{4}\right)E_{A}. \end{array}$$

Let define $\Psi_{1}=\Phi_{1}+\Phi_{2}$, applying Schur complement to solve the bilinear problem in $\Psi_{1}$, we have:

(73) $$\Psi_{1}\leq \Psi_{2}=\left[\begin{array}{ccc} \Phi_{1} & \bar{\Theta } & E_{A}^{T}\\ {}* & \overset{⌢}{R} & 0\\ {}* & * & -{\left(\tau_{1}^{2}R_{1}+\tau_{12}^{2}R_{2}+\frac{\tau_{1}^{2}}{2}R_{3}+\frac{\tau_{12}^{2}}{2}R_{4}\right)}^{-1} \end{array}\right]$$

Pre-multiplying and post-multiplying both sides of $\Psi_{2}$ with $diag\{\underset{18}{\underbrace{I,\ldots ,I}},P\}$, and according to Lemma 4, one has:

(74) $$\Psi_{2}\leq \Psi_{3}=\left[\begin{array}{ccc} \Phi_{1} & \bar{\Theta } & E_{A}^{T}P\\ {}* & \overset{⌢}{R} & 0\\ {}* & * & \left(\tau_{1}^{2}R_{1}+\tau_{12}^{2}R_{2}+\frac{\tau_{1}^{2}}{2}R_{3}+\frac{\tau_{12}^{2}}{2}R_{4}\right)-2P \end{array}\right]$$

It is obvious that $J\left(t\right)<0$, if $\Psi_{3}<0$ is hold, the condition (37) is obtained. Thus, according to the conditions (37) and (38), the following Equation (75) holds:

(75) $$J=\dot{V}\left(t\right)+z{\left(t\right)}^{T}z-\lambda^{2}w^{T}\left(t\right)w\left(t\right)-\kappa \lambda^{2}{\tilde{d}}^{T}\left(t\right)\tilde{d}\left(t\right)<0$$

It can be seen from the condition (75) that the Equation $\dot{V}\left(t\right)<0$ holds in the case of zero-measurement noise ($w\left(t\right)=0$) and zero-estimation error ($\tilde{d}\left(t\right)=0$). This indicates that the asymptotic stability for the system defined in (23) is guaranteed.

Furthermore, integrating both sides of the Equation (75) from zero to any t>0, it follows that

(76) $$V\left(\infty \right)-V\left(0\right)+\int_{0}^{\infty }z^{T}\left(t\right)z\left(t\right)dt<\lambda^{2}\int_{0}^{\infty }w^{T}\left(t\right)w\left(t\right)dt+\kappa \lambda^{2}\int_{0}^{\infty }{\tilde{d}}^{T}\left(t\right)\tilde{d}\left(t\right)dt$$

Considering the system asymptotic stability $V\left(\infty \right)=0$ and the zero-initial condition $V\left(0\right)=0$, we can obtain that the Equation (26) holds. Thus, the H∞ performance of the system (23) can be achieved if the conditions (37) and (38) can be solved. This completes the proof. □

**Remark** **2.** *It can be seen from Theorem 1 that the control gain* K *cannot be directly solved by the LMI technique since the matrix Equation (37) is still a non-convex equation. Meanwhile, considering the uncertainties in the system (23), the original nonlinear matrix inequalities should be transformed into the LMI conditions. Then, the competent conditions solved by the LMI technique have been presented in Theorem 2.*

**Theorem** **2.** *For given scalars*$\tau_{1},\tau_{2},\kappa ,\mu$*, the system (23) with output feedback gain*$K={\left(S^{T}S\right)}^{-1}S^{T}Y$*is asymptotically stable and can satisfy the H∞ performance index (26), if there exist symmetric matrices *$0<P,Q_{i},R_{j}\in \mathbb{R}{\;}^{3\times 3}$*,*i=1,2; j=1,2,3,4*, scalar *λ>0*and any matrices *$N_{k},M_{k},L_{k}\in \mathbb{R}{\;}^{9\times 3}$*,*k=1,2*,*$X\in \mathbb{R}{\;}^{9\times 9}$*,*$Y\in \mathbb{R}{\;}^{3\times 3}$*such that the following matrix inequalities are satisfied:*

(77) $$\left[\begin{array}{ccccccc} {\overset{⌢}{\Phi }}_{1} & \bar{\Theta } & {\overset{⌢}{E}}_{A}^{T} & e_{1}^{T}PE & \mu e_{1}^{T}F^{T} & 0 & \mu e_{1}^{T}F^{T}\\ {}* & \overset{⌢}{R} & 0 & 0 & 0 & 0 & 0\\ {}* & * & \left(\tau_{1}^{2}R_{1}+\tau_{12}^{2}R_{2}+\frac{\tau_{1}^{2}}{2}R_{3}+\frac{\tau_{12}^{2}}{2}R_{4}\right)-2P & 0 & 0 & PE & 0\\ {}* & * & * & -\mu I & 0 & 0 & 0\\ {}* & * & * & * & -\mu I & 0 & 0\\ {}* & * & * & * & * & -\mu I & 0\\ {}* & * & * & * & * & * & -\mu I \end{array}\right]<0$$

(78) $$\left[\begin{array}{cc} \begin{array}{ccc} R_{2}+R_{4} & 0 & 0\\ {}* & 3\left(R_{2}+R_{4}\right) & 0\\ {}* & * & 5\left(R_{2}+R_{4}\right) \end{array} & X\\ {}* & \begin{array}{ccc} R_{2} & 0 & 0\\ {}* & 3R_{2} & 0\\ {}* & * & 5R_{2} \end{array} \end{array}\right]>0$$

where

(79) $$\begin{array}{l} {\overset{⌢}{\Phi }}_{1}=sym\{e_{1}^{T}\left[\begin{array}{cccc} P\bar{A} & YC & YC & PD \end{array}\right]{\bar{E}}_{1}\}+e_{1}^{T}H^{T}He_{1}-e_{11}^{T}\lambda^{2}Ie_{11}-e_{12}^{T}\kappa \lambda^{2}e_{12}+{\bar{E}}_{2}\bar{Q}{\bar{E}}_{2}^{T}\\ -{\left(e_{1}-e_{2}\right)}^{T}R_{1}\left(e_{1}-e_{2}\right)-3{\left(e_{1}+e_{2}-2e_{5}\right)}^{T}R_{1}\left(e_{1}+e_{2}-2e_{5}\right)-5{\left(e_{1}-e_{2}+6e_{5}-6e_{8}\right)}^{T}R_{1}\left(e_{1}-e_{2}+6e_{5}-6e_{8}\right)\\ +\Theta_{1}^{T}\left(N_{1}\Lambda_{1}-N_{2}\Lambda_{2}\right)\Theta_{1}+\Theta_{2}^{T}\left(M_{1}\Lambda_{3}-M_{2}\Lambda_{4}\right)\Theta_{2}+\Theta_{3}^{T}\left(L_{1}\Lambda_{5}-L_{2}\Lambda_{6}\right)\Theta_{3}-{\bar{\zeta }}_{1}^{T}{\bar{R}}_{1}{\bar{\zeta }}_{1}+{\bar{\zeta }}_{1}^{T}{\bar{R}}_{1}{\bar{\zeta }}_{1}, \end{array}$$

(80) $${\overset{⌢}{E}}_{A}=P\bar{A}e_{1}+YCe_{3}+YCe_{11}+PDe_{12}.$$

**Proof.**  In this work, the uncertainties of the motor damping coefficient and driveshaft damping coefficient are considered. Based on the uncertain matrices ΔA defined in the Equation (17), the Equation (37) can be rewritten as follows

(81) $$\left[\begin{array}{ccc} \Phi_{1} & \bar{\Theta } & {\bar{E}}_{A}^{T}P\\ {}* & \overset{⌢}{R} & 0\\ {}* & * & \left(\tau_{1}^{2}R_{1}+\tau_{12}^{2}R_{2}+\frac{\tau_{1}^{2}}{2}R_{3}+\frac{\tau_{12}^{2}}{2}R_{4}\right)-2P \end{array}\right]+sym\{{\bar{e}}_{1}^{T}PEN\left(t\right)F{\bar{e}}_{1}\}+sym\{{\bar{e}}_{2}^{T}PEN\left(t\right)F{\bar{e}}_{1}\}<0$$

where, ${\bar{E}}_{A}=\bar{A}e_{1}+BKCe_{3}+BKCe_{11}+De_{12},\;{\bar{e}}_{1}=\left[\begin{array}{ccc} e_{1} & 0_{18\times 3} & 0_{3\times 3} \end{array}\right],\;{\bar{e}}_{2}=\left[\begin{array}{ccc} 0_{36\times 3} & 0_{18\times 3} & I_{3\times 3} \end{array}\right]$.

By using Lemma 5 and Schur complement, yields

(82) $$\Psi_{3}\leq \Psi_{4}=\left[\begin{array}{ccccccc} \Phi_{1} & \bar{\Theta } & {\bar{E}}_{A}^{T}P & e_{1}^{T}PE & \mu e_{1}^{T}F^{T} & 0 & \mu e_{1}^{T}F^{T}\\ {}* & \overset{⌢}{R} & 0 & 0 & 0 & 0 & 0\\ {}* & * & \left(\tau_{1}^{2}R_{1}+\tau_{12}^{2}R_{2}+\frac{\tau_{1}^{2}}{2}R_{3}+\frac{\tau_{12}^{2}}{2}R_{4}\right)-2P & 0 & 0 & PE & 0\\ {}* & * & * & -\mu I & 0 & 0 & 0\\ {}* & * & * & * & -\mu I & 0 & 0\\ {}* & * & * & * & * & -\mu I & 0\\ {}* & * & * & * & * & * & -\mu I \end{array}\right]$$

Define Y=PBK, PB=S, one has $K=S^{-1}Y={\left(S^{T}S\right)}^{-1}S^{T}Y$, then the matrix $\Psi_{4}$ can be rewritten as:

(83) $$\Psi_{4}=\left[\begin{array}{ccccccc} {\overset{⌢}{\Phi }}_{1} & \bar{\Theta } & {\overset{⌢}{E}}_{A}^{T} & e_{1}^{T}PE & \mu e_{1}^{T}F^{T} & 0 & \mu e_{1}^{T}F^{T}\\ {}* & \overset{⌢}{R} & 0 & 0 & 0 & 0 & 0\\ {}* & * & \left(\tau_{1}^{2}R_{1}+\tau_{12}^{2}R_{2}+\frac{\tau_{1}^{2}}{2}R_{3}+\frac{\tau_{12}^{2}}{2}R_{4}\right)-2P & 0 & 0 & PE & 0\\ {}* & * & * & -\mu I & 0 & 0 & 0\\ {}* & * & * & * & -\mu I & 0 & 0\\ {}* & * & * & * & * & -\mu I & 0\\ {}* & * & * & * & * & * & -\mu I \end{array}\right]$$

If $\Psi_{4}<0$ is satisfied, the Equation (81) holds. Thus, the condition (77) can be obtained. The Equation (78) is equivalent to the condition (38). This completes the proof. □

**Remark** **3.** *By solving Theorem 2, the corresponding vehicle speed tracking control gain can be obtained. As mentioned in Section 1, many advanced control methods (such as MPC and SMC) have been proposed to achieve speed tracking control during one of the different disturbances. From the perspective of engineering application, these advanced methods may be complex and have a large number of calculations for the in-vehicle controller under simultaneously considering different disturbances. Thus, this study chooses the PID and traditional H∞ state feedback control algorithms for comparative purposes, which are easy to be practically applied to verify the control effect in future experiments. To demonstrate the effectiveness of the proposed controller, the PID controller and the traditional H∞ state feedback controller with commutation delay are introduced in the following section.*

## 5. Results and Discussions

### 5.1. Parameters of Vehicle Model and Controller

**Figure 2:**
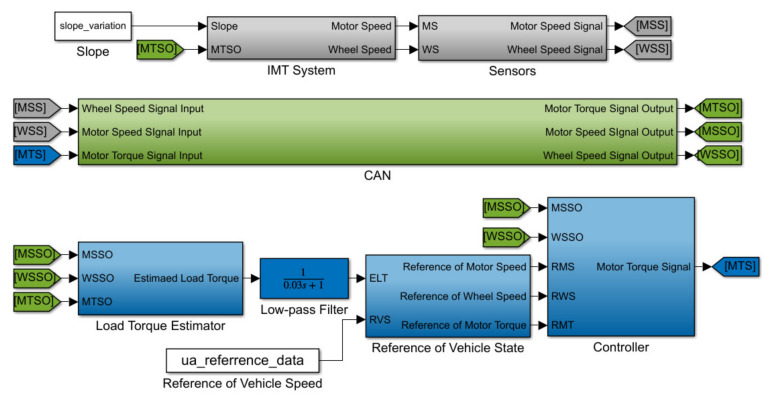
Simulation model for IMT control system.

**Figure 3:**
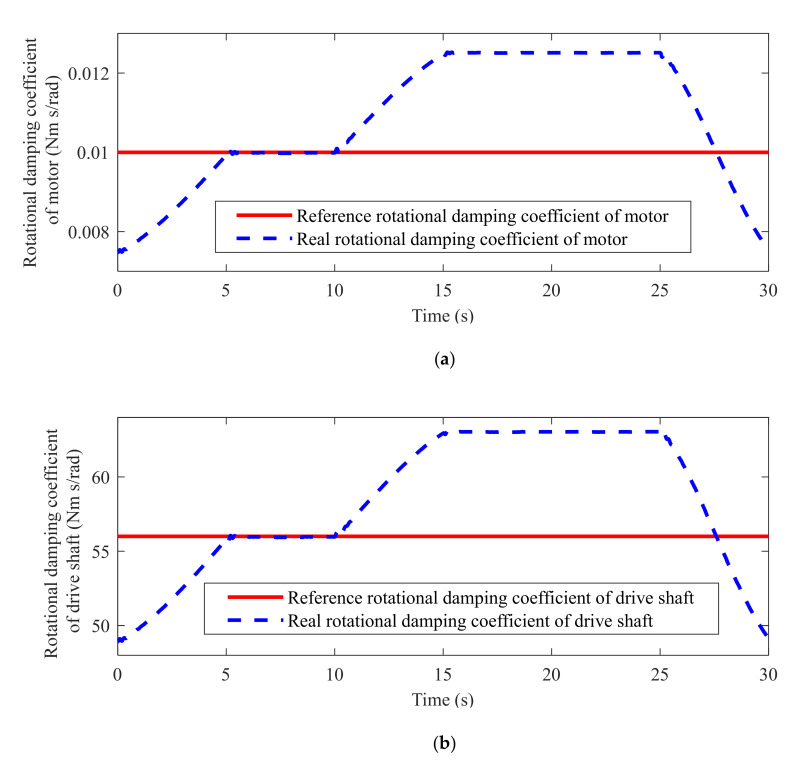
Rotational damping coefficient of (**a**) motor (**b**) drive shaft.

The proposed method is employed to design the robust speed tracking controller for the IMT powertrain system to achieve the required tracking performance under non-ideal working conditions. Based on the schematic diagram in Figure 1, the designed simulation platform is established in Figure 2 to evaluate the proposed controller. As shown in Figure 2, the entire system is composed of an IMT powertrain system module, a sensors module, a CAN module, a load torque estimator, a low-pass filter module, a vehicle state reference module, and a controller module. The IMT powertrain system module receives the motor torque command from the CAN unit and computes the corresponding motor speed and wheel speed. The actual vehicle speed in the simulation is calculated as the wheel speed times the wheel radius. The relevant physical and uncertain parameters of the IMT powertrain system can be found in [33,40], which are listed in Table 1. As shown in Figure 3, the parametric uncertainties of shaft damping and motor damping coefficients are assumed to be 15% and 30% of their nominal value, respectively [40].

**Figure 4:**
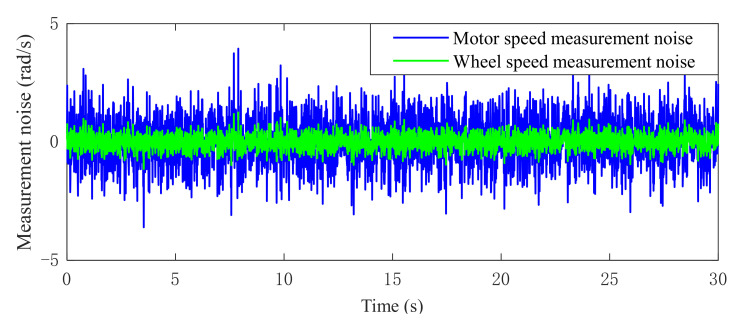
Measurement noise in sensors.

Moreover, the motor speed and wheel speed can be measured by the sensor module. To construct the non-ideal working condition, the band-limited white noises with different noise powers are used as the measurement noises for the motor speed signal and wheel speed signal, which are shown in Figure 4, respectively.

**Figure 5:**
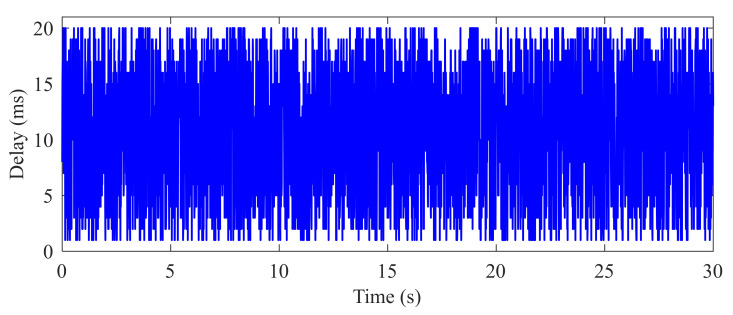
Time-varying delay in signal transmission.

The CAN module is conducted to generate the CAN-induced delay. Based on the published works [22,34], it can be found that the length of CAN-induced delay has an approximately linear relationship with the signal priorities. Thus, a simplified and conservative definition of CAN-induced delay distribution is introduced in this application study to investigate the robustness of the proposed controller. As shown in Figure 5, the lower bound of the CAN-induced delay is set as 1 ms and the upper bound is set as 20 ms. In the simulation cases, the CAN-induced delay shown in Figure 5 is time-varying and conservatively assumed to be uniformly distributed in the corresponding interval. Furthermore, the load torque observer is employed to estimate the external load torque by the signals of motor torque, motor speed and wheel speed from the CAN unit. The external load torque to be estimated is usually of low frequency, whereas the measurement noise is of medium or high frequency. To reduce the estimation error introduced by the measurement noises, a first-order system is employed as the low-pass filter [32], which is written as:

(84) $$F\left(s\right)=\frac{1}{Ts+1}$$

where T is the time constant of the system, which is determined T=0.03 s by the trial and error method.

Finally, the references of vehicle state are calculated by the state reference module and the target motor torque can be solved by the controller module.

Taking into account the motor maximum torque, the saturation functions are employed to restrict the motor torque, motor speed, and motor power. They are written as

(85) $$\begin{array}{l} -340\;\mathrm{Nm}\leq T_{m}\leq 340\;\mathrm{Nm},\\ -2500\;\mathrm{Nm}/s\leq {\dot{T}}_{m}\leq 2500\;\mathrm{Nm}/s,\\ 0<w_{m}<9000\;r/\min. \end{array}$$

To prove the advantage of the proposed robust speed tracking controller (RSTC), the PID controller (PIDC) and the H∞ state feedback controller (SFC) with commutation delays are comprehensively compared. By setting the positive scalars $\tau_{1}=1$ ms, $\tau_{2}=20$ ms, κ=1 and μ=0.007, it can be obtained that the minimum guaranteed closed-loop system H∞ performance index is calculated as $\gamma_{\min}=14.4345$ and the admissible control gain can be obtained by solving Theorem 2. Meanwhile, the admissible control gain of the SFC with H∞ performance index $\gamma_{\min}=130.3630$ is calculated by the free-weighting matrix method [23], and the admissible control gain of the PIDC is determined by the trial-and-error method. The corresponding control gains of the three controllers are also provided in Table 2. The maximum speed tracking errors of the RSTC are smaller than the PIDC and the SFC though there exists the road slope variation.

### 5.2. Performance Analysis of Robust Speed Tracking Controller

#### 5.2.1. Simulation Conditions

**Figure 6:**
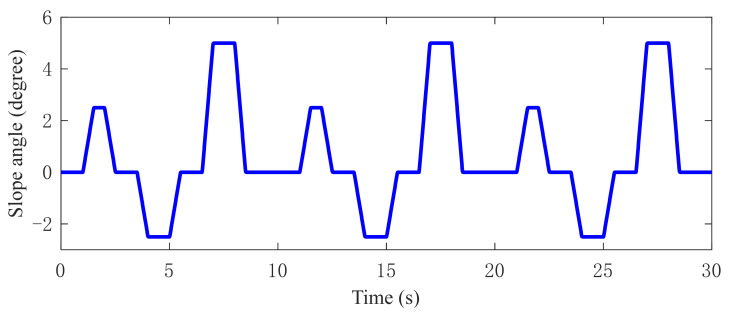
Unknown road slope angle variation.

In the simulation, a target speed curve is designed. The speed curve includes the typical acceleration test, deceleration test, and constant velocity test, in which the regular driving conditions are considered. In the designed speed curve, to verify the controller robustness under the speed variation, the acceleration is set as 1.67 m/s^2^ and the deceleration is set as 3.33 m/s^2^, and both are higher than the maximum acceleration of 1.11 m/s^2^ and maximum deceleration of 2.22 m/s^2^ in the New European Driving Cycle (NEDC). The designed constant velocity is 30 km/h and 60 km/h, both of which are typical vehicle cruise speeds. The time-varying road gradient is given in Figure 6.

**Figure 7:**
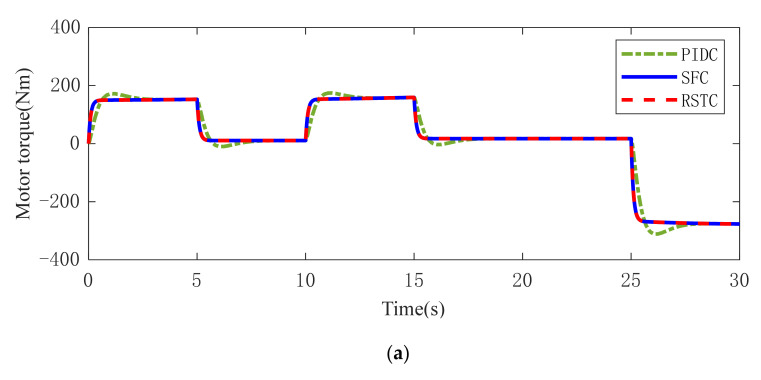

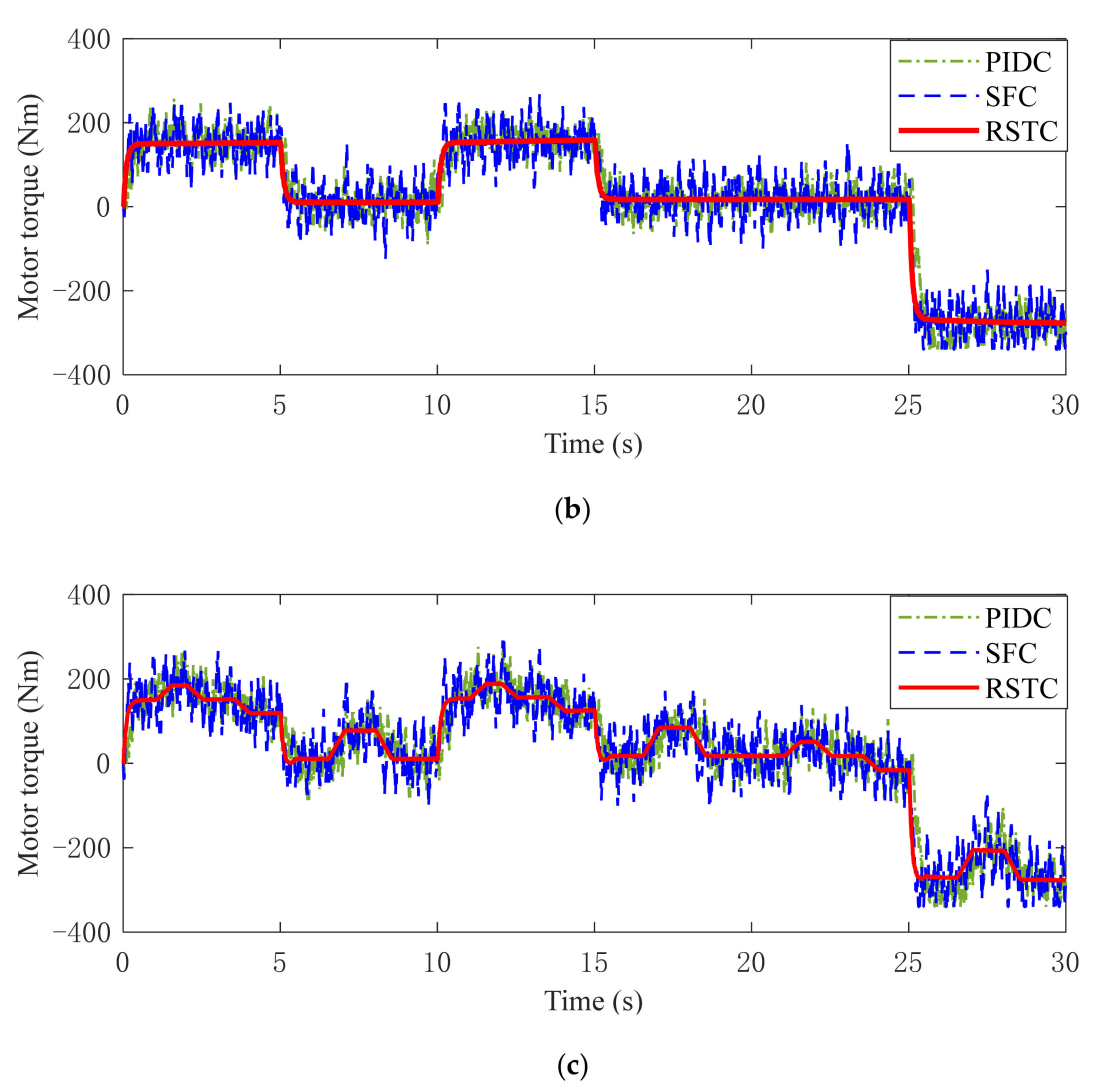
Motor torque dynamic response in (**a**) Condition 1 (**b**) Condition 2 (**c**) Condition 3.

**Figure 8:**
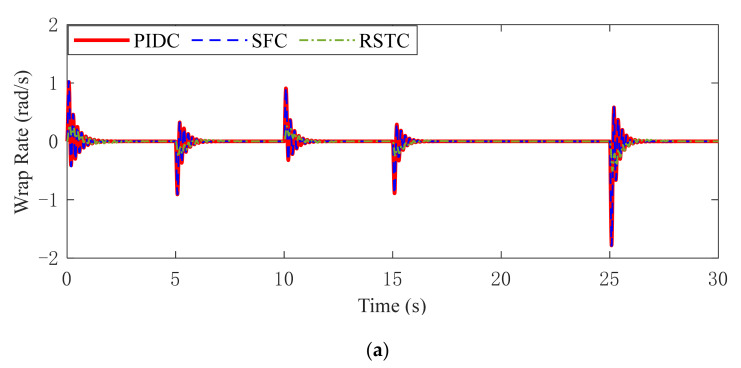

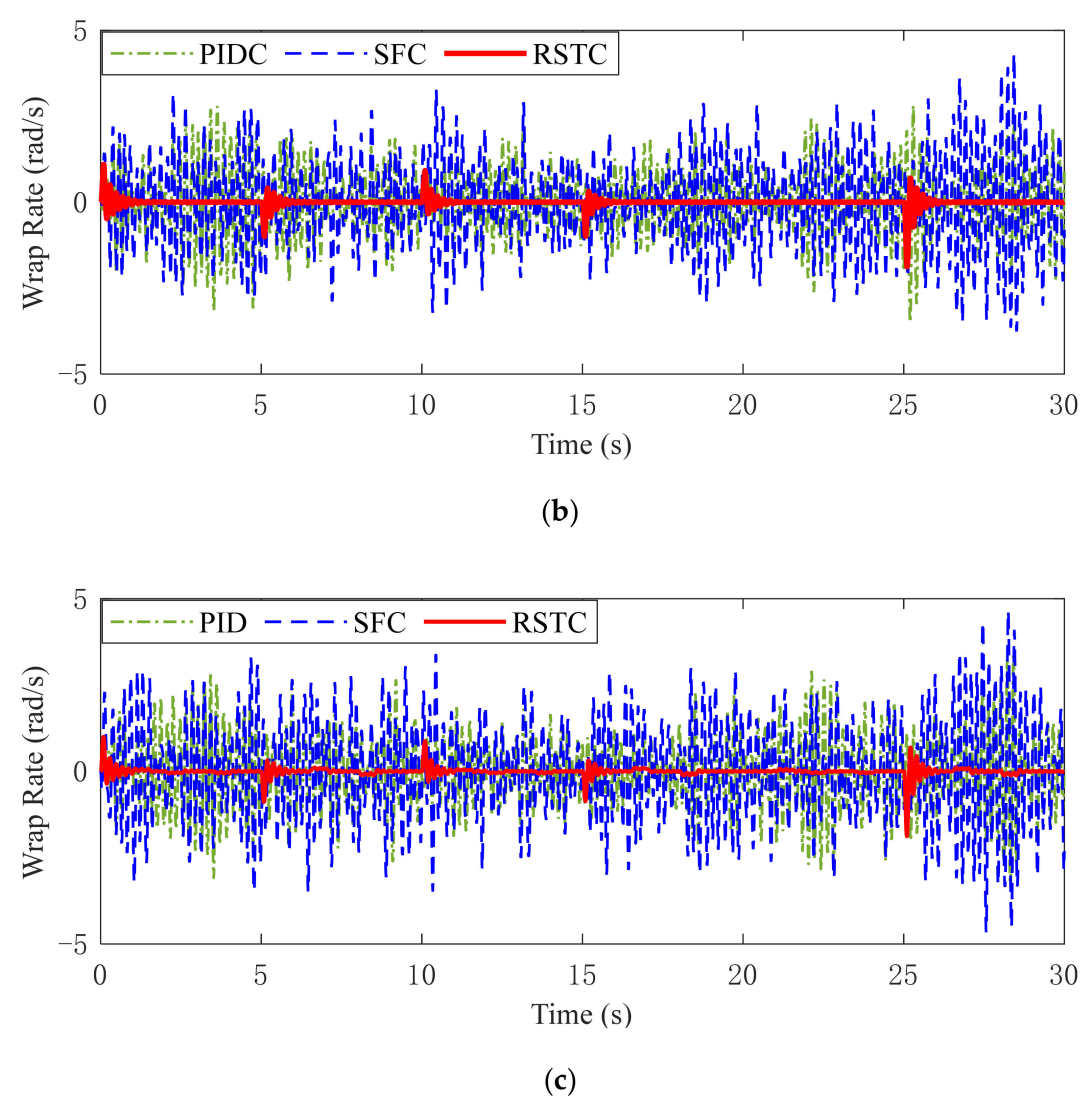
Wrap rate dynamic responses in (**a**) Condition 1 (**b**) Condition 2 (**c**) Condition 3.

**Figure 9:**
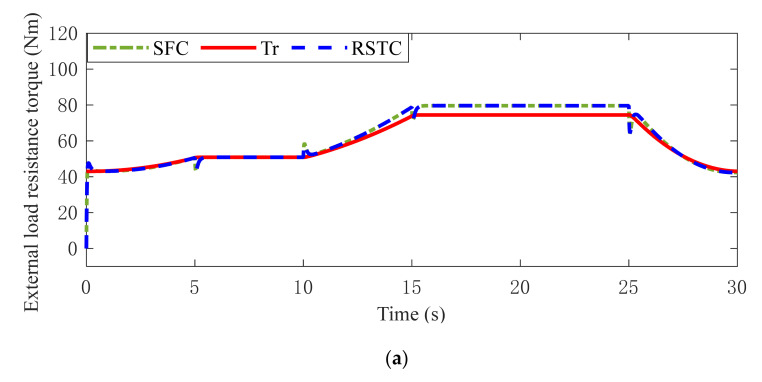

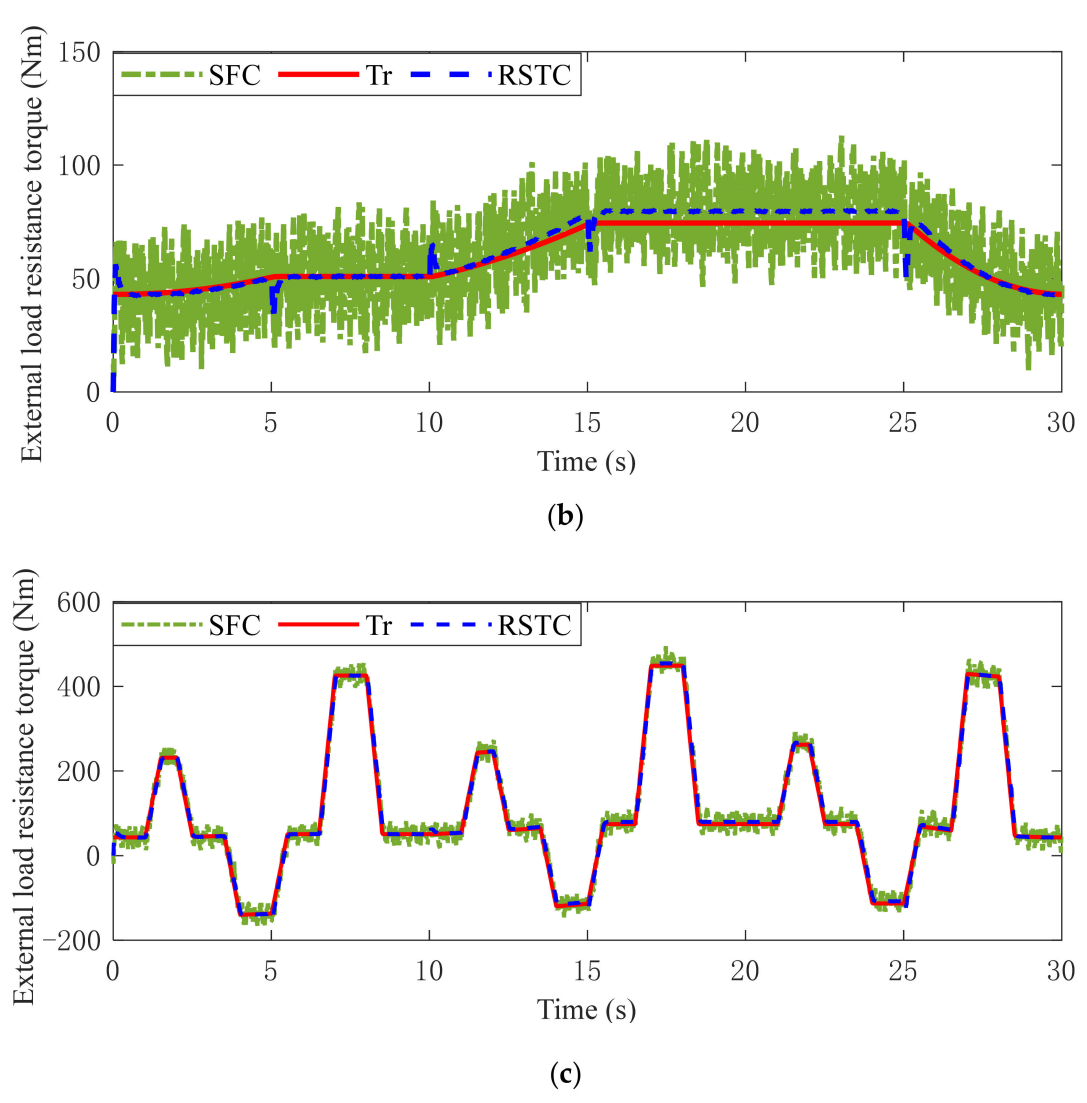
Estimated road resistance responses in (**a**) Condition 1 (**b**) Condition 2 (**c**) Condition 3.

**Figure 10:**
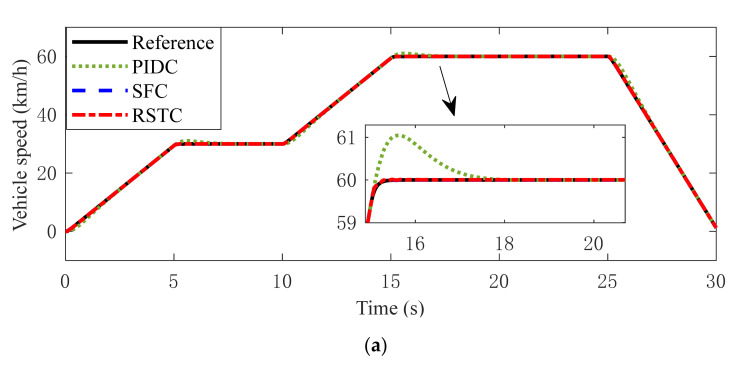

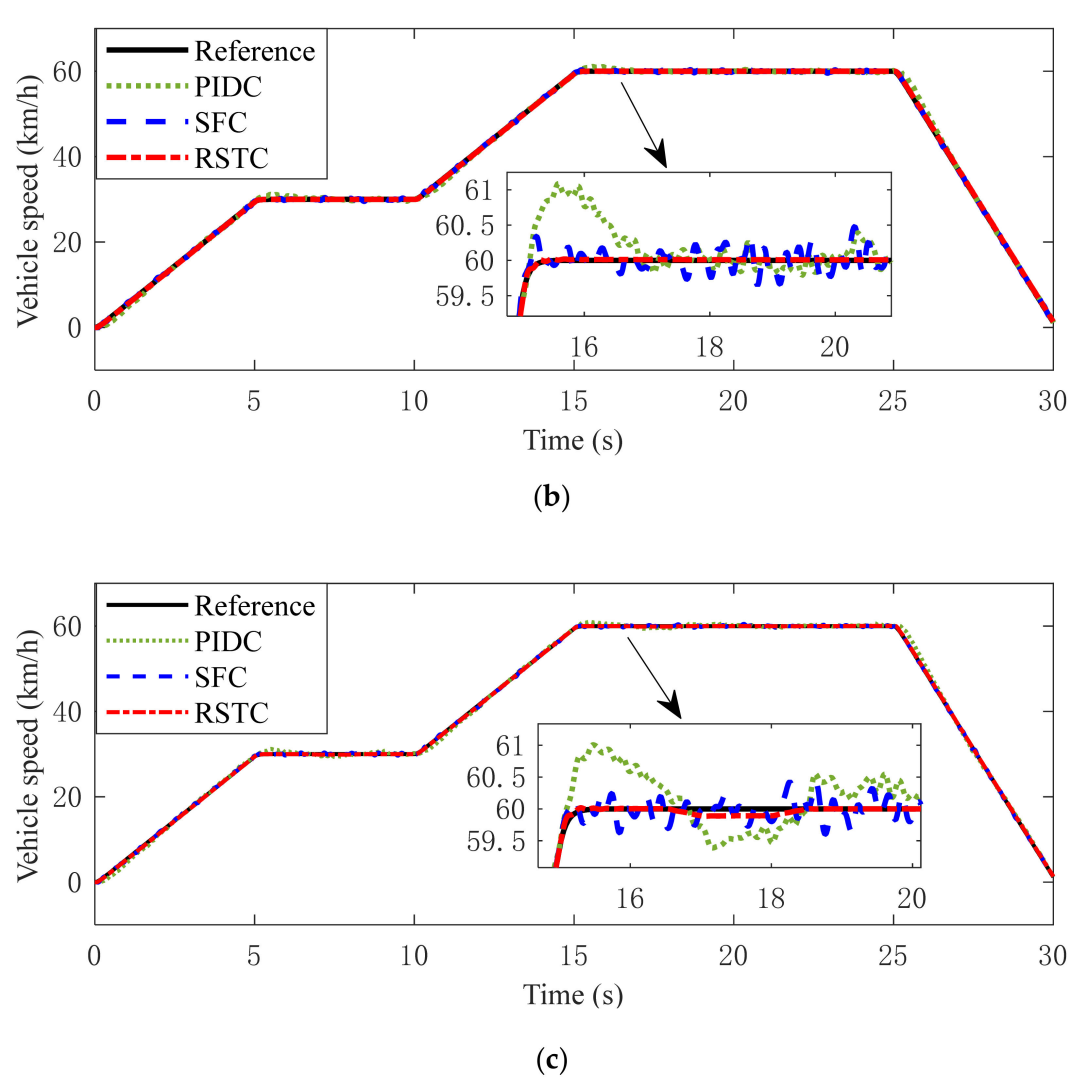
Vehicle speed tracking responses in (**a**) Condition 1 (**b**) Condition 2 (**c**) Condition 3.

To analyze the effect of different interference factors on the performance of vehicle speed tracking, three interference conditions are considered in this work. For Condition 1, only the uncertainty interference of the motor and drive shaft rotational damping coefficient is introduced. Since both the measurement noise and signal transmission time delay exist during the signal transmission process, they are introduced to the simulation simultaneously. For Condition 2, the parameter uncertainty, measurement noise and signal transmission time delay are employed. Compared with Condition 2, the unknown road slope angle variation in Figure 6 is also considered in Condition 3. The simulation results under interference different conditions are shown in Figure 7, Figure 8, Figure 9 and Figure 10.

#### 5.2.2. Performance Comparisons

The motor torque output under three controllers is plotted in Figure 7 and the standard deviations of motor torque are listed in Table 3. It is well known that the large torque variation can generate strong motor torque oscillation. To evaluate the torque variation range, the dynamic torque responses between 15 s and 25 s are used to calculate the standard deviation. In Condition 1, the motor torque under the three controllers is stable. In Condition 2, the motor torque under PIDC or SFC is jagged. In practice, the torque oscillation should be avoided, as it would increase the motor load and powertrain jitter. However, RSTC makes the motor torque more stable, which indicates that the oscillation is greatly suppressed under time delay and measurement noise. In Condition 3, the motor torque under PIDC or SFC is jagged greatly, which is similar to the torque in Figure 7b. It can be found that RSTC generates smooth torque output even there are a time delay and measurement noise.

The shaft wrap rate, the difference between the motor rotation speed divided by the gear ratio and the wheel rotation speed, is utilized to represent the jitter of the vehicle driveline. The wrap rate under three controllers is shown in Figure 8 and the standard deviations of wrap rate are listed in Table 4. In Condition 1, the wrap rate under three controllers only fluctuates significantly when the motor torque changes rapidly, and the wrap rate under RSTC has the smallest fluctuation amplitude. In Condition 2, the wrap rate responses under PIDC or SFC controller oscillate obviously during the simulation running. By contrast, RSTC can reduce the fluctuation of wrap rate when the motor torque changes quickly. It indicates that drive axle durability and passenger ride comfort are improved compared with PIDC and SFC. In Condition 3, the wrap rates under PIDC and SFC have higher amplitude than RSTC. Due to the extra resistance change caused by the road slope variation, the wrap rates in Condition 3 have greater oscillation than without road slope variation in Condition 2.

To describe the external disturbance induced by the unknown road condition, the road resistance is estimated by Equation (10). The estimated road resistance in Condition 1 is presented in Figure 9a, in which the legend Tr means the real road resistance in Equation (6), the legend SFC means the estimated road resistance under SFC, and the RSTC means the estimated road resistance under RSTC. Since the elasticity of the driving shaft is neglected in the Equation (10), it can be seen that the error of resistance torque estimation increases when the axle wrap rate oscillates. Owing to the parameter uncertainties of IMT powertrain system, there is a small estimation error during the whole driving process. To improve the anti-disturbance ability, the estimation error is considered in the robust controller design.

As shown in Figure 9b,c, the road resistance is estimated and processed by the low-pass filter. It can be found in Figure 9a,b that the estimated resistance torque under SFC fluctuates greatly due to the time delay and measurement noise. The estimated resistance torque under RSTC fluctuates slightly when the axle wrap rate oscillates. Similarly, it can be seen from Figure 9c that the road torque estimated by SFC tracks the real external resistance torque with the obvious fluctuation. But, the estimator with RSTC can ensure a stable estimation performance, except for the slight fluctuation when the axle wrap rate changes sharply. The reason is that the motor torque output and the axle wrap rate under RSTC are more stable than SFC. It is easy to obtain from the Equation (10) that the smaller axle wrap rate and more stable motor torques can achieve a more accurate and stable estimation. Moreover, it is worth noting that the estimated Tr in Equation (6) includes not only the road resistance but also the air resistance. Thus, the disturbance caused by rolling resistance coefficient variation and unknown wind speed is also precisely estimated.

The maximum values and standard deviations of road resistance estimation error are given in Table 5. It can be seen that both the maximum values and standard deviations of road resistance estimation error under RSTC are less than under SFC in Condition 2 and Condition 3, which means that RSTC has the more accurate and stable estimation performance.

The speed tracking performance comparison under three controllers is shown in Figure 10. In Condition 1, all three controllers can quickly track the reference speed. But, the speed response under PIDC generates a slight overshoot. It can be found in Figure 10a,b that the vehicle speed under RSTC control is more stable than under PIDC and SFC with the measurement noise and signal transmission time delay. It can be seen in Figure 10b,c that the vehicle speed under PIDC and SFC suffers a greater fluctuation owing to the road slope variation. The RSTC makes the vehicle speed accurately track the target speed, except for a slight tracking error when road slope varies. Therefore, the conclusion can be drawn that the RSTC has better robustness to the time delay, measurement noise, road slope variation and parameter uncertainties than the PIDC and SFC.

**Figure 11:**
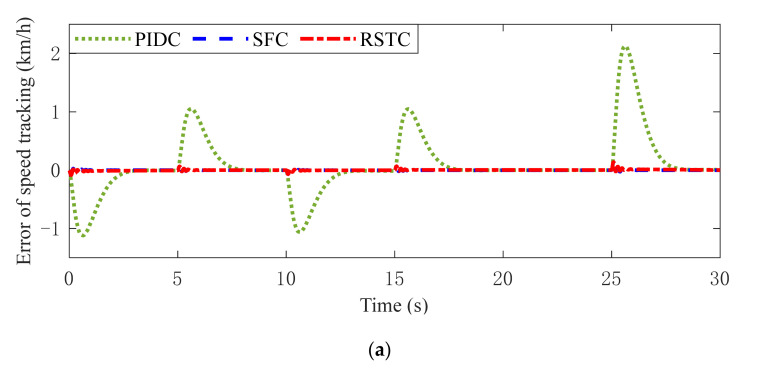

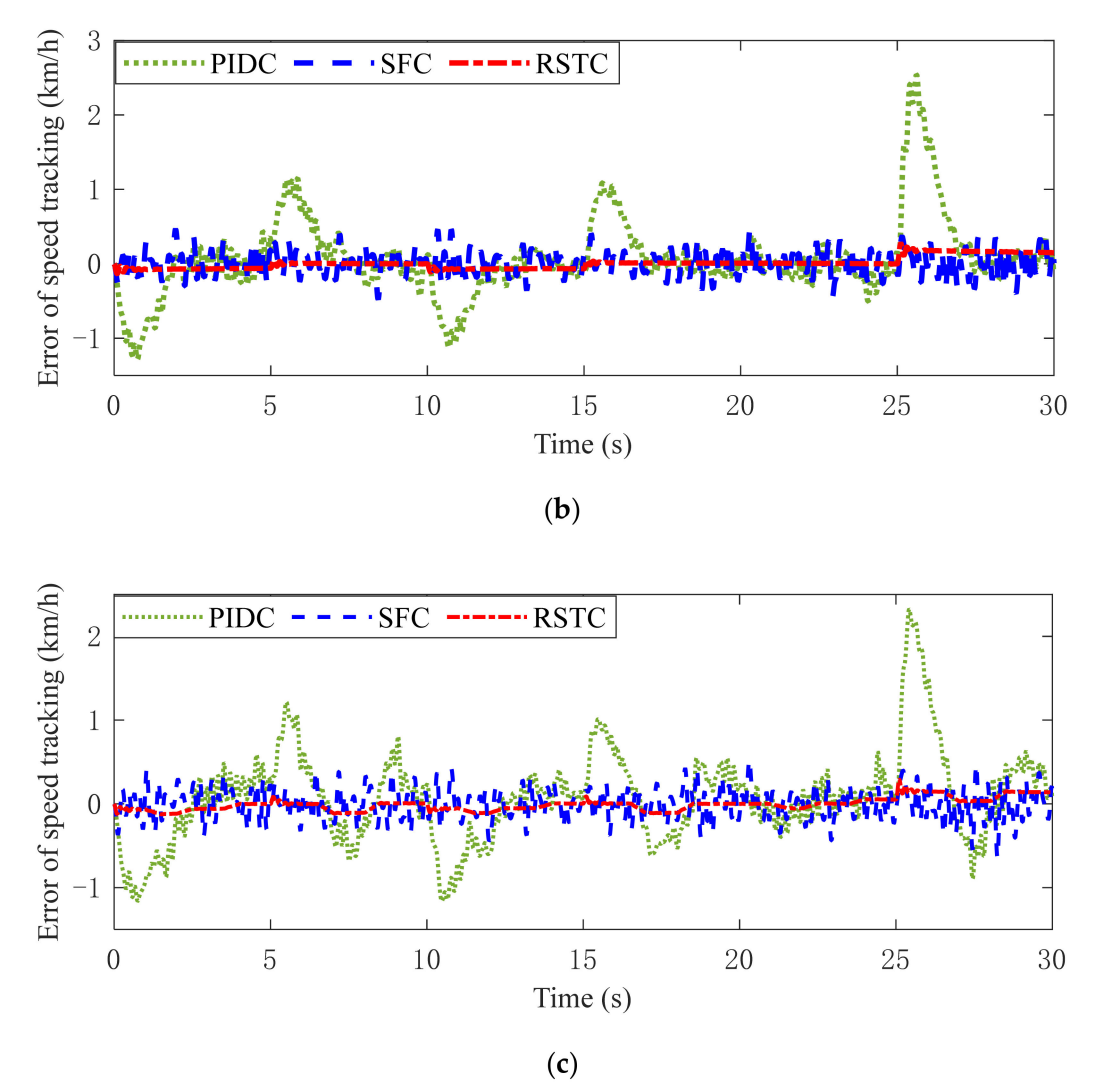
Vehicle speed tracking error responses in (**a**) Condition 1 (**b**) Condition 2 (**c**) Condition 3.

To further verify the speed tracking performance of the proposed controller, the speed tracking errors are shown in Figure 11. It can be found in Figure 11a that the PIDC has the larger tracking error with the wrap rate variation at about 0 s, 5 s, 10 s, 15 s and 25 s due to the torsion of the flexible drive shaft. In Condition 2, compared with the PIDC and SFC, RSTC keeps the tracking error in a minimum range without fluctuation. The tracking error standard deviations of the PIDC, RHC and RSTC are 0.5548 km/h, 0.1503 km/h and 0.0625 km/h, respectively. The maximum tracking error of the PIDC, SFC and RSTC is 2.3954 km/h at 25.63 s, 0.5840 km/h at 10.31 s, and 0.2056 km/h at 25.11 s, respectively. Figure 11c shows the tracking errors of different controllers under road slope variation. Due to the stable motor torque output, RSTC keeps the tracking error in a minimum range. Their standard deviations are 0.5600 km/h, 0.1522 km/h and 0.0766 km/h, respectively. The maximum tracking errors of the PIDC, RHC and RSTC are 2.2188 km/h at 25.65 s, 0.5133 km/h at 28.46 s and 0.2734 km/h at 25.11 s, respectively. These results demonstrate that the RSTC has better speed tracking ability and accuracy than the PIDC and RSTC.

**Remark** **4.** Although this work copes with different disturbances in controller design to improve the speed tracking ability, there are still methodological limitations in the proposed control method. Considering the cost, the speed is usually calculated by the wheel speed in engineering applications. Thus, this study designs the speed controller based on this assumption, which is not valid when the wheels slip. The speed should be further estimated by combining the acceleration sensor or GPS. Then, the CAN-induced delay is assumed to be uniformly distributed. This assumption is conservative given that it can be nonstationary in real driving conditions. Thus, the nonstationary condition of CAN-induced delay should be further investigated. Moreover, the stiffness coefficient and damping coefficient of the IMT powertrain system may be nonlinear, thus the linear assumption seems to be conservative.

## 6. Conclusions

This paper proposed a robust speed tracking control method for future EVs with IMT powertrain system to achieve precise speed tracking performance. Compared with the two implemented control methods (PIDC and SFC), the main contributions of this study are summarized as follows:

(1) The network-induced delay, parameter uncertainties, measurement noise and unknown road slope variation are simultaneously considered to improve the robustness of the speed tracking controller. There are a few works comprehensively addressing these control issues.
(2) The road resistance observer is synthesized in the speed tracking controller design, which can timely respond to the road slope variation. Thus, the proposed control method can guarantee a smaller overshoot of the dynamic responses.
(3) A novel Lyapunov function is proposed in the speed tracking controller design to ensure the global asymptotic stability conditions with the required H∞ performance. This can reduce the possible design conservativeness and improve the controller robustness.

In further works, the nonstationary distribution of CAN-induced delay and nonlinear property of the IMT powertrain system should be analyzed, and more experiments should be conducted under different disturbances to further verify the effectiveness of the proposed method. Moreover, the advanced control methods would be investigated to further improve the controller robustness.

## Figures and Tables

**Table 1:** Numerical parameters of IMT powertrain system.

$J_{n}$	Composite inertia of motor and gearbox	0.31 kg·m^2^
$J_{w}$	Inertia of wheels	1.7747 kg·m^2^
$m_{v}$	Vehicle mass	1463 kg
$r_{w}$	Wheel radius	0.3 m
$k_{s}$	Shaft spring coefficient	9520 Nm/rad
$c_{s}$	Shaft damping coefficient	56 ± (56 × 15%) Nm·s/rad
$i_{a}$	Gear ratio	5.59
$\rho_{air}$	Air density	1.29 kg/m^3^
$C_{d}$	Aerodynamic drag coefficient	0.325
$A_{f}$	Effective front area	1.8 m^2^
g	Gravitational acceleration	9.8 m/s^2^
μ	Tire rolling resistance coefficent	0.01 Nm·s/rad
$c_{m}$	Motor damping coefficient	0.01 ± (0.01 × 30%) Nm·s/rad

**Table 2:** Gains and performance index of controllers.

	Index	Gain	Performance Index
Controllers			
PIDC		$K_{P}=-98.3\;;K_{I}=-117.8;\;K_{D}=-1.4$	None
SFC [23]		$K=\left[\begin{array}{cc} -3.7189 & -286.5371 \end{array}\right]$	$\gamma_{\min}=130.3630$
RSTC		$K=\left[\begin{array}{cc} -0.0188 & -1.1646 \end{array}\right]$	$\gamma_{\min}=14.4345$

**Table 3:** Standard deviations of motor torque between 15 s and 25 s.

	Controllers	PIDC	SFC	RSTC
Conditions				
Condition 1		20.8652 Nm	11.6532 Nm	11.5835 Nm
Condition 2		35.3368 Nm	42.9183 Nm	12.5615 Nm
Condition 3		44.2241 Nm	51.7989 Nm	28.0982 Nm

**Table 4:** Standard deviations of wrap rate.

	Controllers	PID	SFC	RSTC
Conditions				
Condition 1		0.0598 rad/s	0.1379 rad/s	0.1363 rad/s
Condition 2		0.9715 rad/s	1.2878 rad/s	0.1379 rad/s
Condition 3		1.0304 rad/s	1.4069 rad/s	0.1449 rad/s

**Table 5:** Maximum values and standard deviations of road resistance estimation error.

	Conditions	SFC	RSTC
Controllers			
Condition 1	Maximum error	7.5929 Nm	7.4880 Nm
	Standard deviations of error	2.8175 Nm	2.8157 Nm
Condition 2	Maximum error	38.4857 Nm	13.9597 Nm
	Standard deviations of error	14.6416 Nm	3.2688 Nm
Condition 3	Maximum error	59.7465 Nm	28.1914 Nm
	Standard deviations of error	17.3692 Nm	9.2753 Nm

## Data Availability

Not applicable.
